# Intratumoral and fecal microbiota reveals microbial markers associated with gastric carcinogenesis

**DOI:** 10.3389/fcimb.2024.1397466

**Published:** 2024-09-17

**Authors:** Yiwen Wang, Yue Wang, Wenjie Han, Mengzhen Han, Xiaolin Liu, Jianying Dai, Yuesheng Dong, Tao Sun, Junnan Xu

**Affiliations:** ^1^ Department of Breast Medicine 1, Cancer Hospital of China Medical University, Liaoning Cancer Hospital, Shenyang, China; ^2^ Department of Pharmacology, Cancer Hospital of China Medical University, Liaoning Cancer Hospital, Shenyang, China; ^3^ Department of Bioinformatics, Kanghui Biotechnology Co., Ltd., Shenyang, China; ^4^ School of Bioengineering, Dalian University of Technology, Dalian, Liaoning, China; ^5^ Department of Oncology Medicine, Key Laboratory of Liaoning Breast Cancer Research, Shenyang, Liaoning, China; ^6^ Department of Breast Medicine, Cancer Hospital of Dalian University of Technology, Liaoning Cancer Hospital, Shenyang, China

**Keywords:** gastric cancer, intratumoral microbiota, fecal microbiota, microbial marker, non-invasive prediction

## Abstract

**Background:**

The relationship between dysbiosis of the gastrointestinal microbiota and gastric cancer (GC) has been extensively studied. However, microbiota alterations in GC patients vary widely across studies, and reproducible diagnostic biomarkers for early GC are still lacking in multiple populations. Thus, this study aimed to characterize the gastrointestinal microbial communities involved in gastric carcinogenesis through a meta-analysis of multiple published and open datasets.

**Methods:**

We analyzed 16S rRNA sequencing data from 1,642 gastric biopsy samples and 394 stool samples across 11 independent studies. VSEARCH, QIIME and R packages such as vegan, phyloseq, cooccur, and random forest were used for data processing and analysis. PICRUSt software was employed to predict functions.

**Results:**

The α-diversity results indicated significant differences in the intratumoral microbiota of cancer patients compared to non-cancer patients, while no significant differences were observed in the fecal microbiota. Network analysis showed that the positive correlation with GC-enriched bacteria increased, and the positive correlation with GC-depleted bacteria decreased compared to healthy individuals. Functional analyses indicated that pathways related to carbohydrate metabolism were significantly enriched in GC, while biosynthesis of unsaturated fatty acids was diminished. Additionally, we investigated non-*Helicobacter pylori (HP)* commensals, which are crucial in both *HP*-negative and *HP*-positive GC. Random forest models, constructed using specific taxa associated with GC identified from the LEfSe analysis, revealed that the combination of Lactobacillus and Streptococcus included alone could effectively discriminate between GC patients and healthy individuals in fecal samples (area under the curve (AUC) = 0.7949). This finding was also validated in an independent cohort (AUC = 0.7712).

**Conclusions:**

This study examined the intratumoral and fecal microbiota of GC patients from a dual microecological perspective and identified *Lactobacillus, Streptococcus, Roseburia, Faecalibacterium* and *Phascolarctobacterium* as intratumoral and intestinal-specific co-differential bacteria. Furthermore, it confirmed the validity of the combination of *Lactobacillus* and *Streptococcus* as GC-specific microbial markers across multiple populations, which may aid in the early non-invasive diagnosis of GC.

## 1 Introduction

Gastric cancer (GC) ranks as the fifth most common cancer and the fourth leading cause of cancer deaths globally (Yang et al., 2023). While GC’s incidence and mortality rates have declined over recent decades, in China, GC remains the third most prevalent and deadly among all malignant tumors (Huang et al., 2023; Yang et al., 2023). Conversely, in Japan and South Korea, where GC also presents high incidence rates, significant reductions in mortality have been achieved through widespread endoscopic screening, which facilitates the early detection of GC (Namasivayam, 2023). This fact highlights the significance of early screening. Currently, the gold standard for early diagnosis of GC involves endoscopy and biopsy, both costly and invasive techniques that have resulted in low screening acceptance (Mejía-Guarnizo et al., 2023). Consequently, there is a pressing need for more precise, accessible, low-cost, and non-invasive biomarkers to assist in the early diagnosis of GC and monitoring for relapse.

*Helicobacter pylori* (*HP*) is classified as a class I carcinogen for GC (Weng et al., 2019; Yang et al., 2021). Effective eradication of *HP* can significantly reduce the risk of developing GC (Li et al., 2023; Yarahmadi and Afkhami, 2024). However, eradicating *HP* does not entirely prevent the development of GC (Cheung and Leung, 2018). Many studies have observed that as *HP*-positive GC progresses, the relative abundance of *HP* tends to decrease, concomitant with an increase in the relative abundance of some other bacteria (Ferreira et al., 2018; Hsieh et al., 2018; Liu et al., 2022a). Additionally, *HP*-negative GC constitutes approximately 0.42% to 5.4% of all GC cases (Yamamoto et al., 2015). Thus, other gastric microorganisms apart from *HP* may also play roles in gastric carcinogenesis. Lertpiriyapong et al. observed that *HP* mice colonized with complex or restricted microbiota were more susceptible to developing GC than germ-free and *HP*-monoassociated mice (Lertpiriyapong et al., 2014). Furthermore, even in the absence of *HP* infection, three commensal bacteria have been shown to induce gastritis and dysplasia in mice (Lertpiriyapong et al., 2014). These findings suggest that non-*HP* microorganisms may contribute to the development of GC, either alongside or independent of *HP*, necessitating further investigation.

With the continuous advancement of microbial analysis techniques and methods, many independent studies employing 16S ribosomal RNA (rRNA) sequencing have revealed changes in the gastric microbiota of non-GC populations and GC patients (Castaño-Rodríguez et al., 2017; Yu et al., 2017; Coker et al., 2018; Chen et al., 2019; Peng et al., 2023). Notably, the intestinal microbiota from GC patients, a critical source of gastric microbiota, also exhibits changes (Qi et al., 2019; Wu et al., 2020; Liu et al., 2021b; He et al., 2022b). Microbial markers based on fecal samples offer more accessible and non-invasive alternatives than those based on gastric tissue samples. Nevertheless, the reports of changes in intratumoral and fecal microbiota in GC are inconsistent across studies, likely due to variations in subjects’ age and gender, geographic locations, and sequencing techniques. Additionally, few studies have focused on predicting GC by microbial markers based on fecal samples, and the reproducibility and accuracy of these markers remain uncertain. Hence, a comprehensive multi-cohort analysis is necessary to minimize the interference of various confounding factors and establish consistency across multiple studies (Wu et al., 2021). In this study, we integrated and reanalyzed 16S rRNA sequencing data from 1,642 gastric biopsy samples and 394 stool samples across 11 independent studies. We detailed changes in the composition and taxonomic classification of the gastrointestinal microbiota during GC progression. Differences in associated networks and functions between healthy individuals and GC patients in gastric tissue samples were investigated. Moreover, we characterized the microbiota changes associated with developing of *HP*-negative and *HP*-positive GC. Finally, microbial markers capable of distinguishing GC patients from healthy individuals were identified and validated. Our goal is to combine the intratumoral microbiota and fecal microbiota of GC patients in a meta-analysis so as to explore the key differential flora during gastric carcinogenesis from a dual microecological perspective and to provide a new method for early screening of GC by constructing random forest models.

## 2 Methods

### 2.1 Literature search and study selection

An exhaustive literature search was conducted on August 9, 2023, utilizing PubMed, Web of Science, and Embase databases. Detailed search formulas are documented in (**Supplementary Table S1**). The language was restricted to English, and types such as reviews, meta-analyses, and case reports were excluded. Two authors independently reviewed the title and abstract of each study, and the full text was retrieved if the abstract content warranted further examination. Additional manual searches of the reference lists were conducted to ensure a thorough literature search. The selected studies had to meet the following criteria: (1) raw gene sequencing data could be downloaded and grouped; (2) used fecal or gastric tissues as samples; (3) included GC and normal or benign controls. However, patients who had undergone radiotherapy or chemotherapy or had been treated with antibiotics or probiotics within the past month were excluded.

### 2.2 Data acquisition and processing

The accession number (BioProject ID) was entered into the Sequence Read Archive (SRA) database to download the sequencing and biosample data. After the FASTQ files were extracted, they were de-multiplied. Raw data were integrated using VSEARCH software (v2.18.0) to cut out primers and barcodes and filter low-quality data (Rognes et al., 2016). After removing duplicates and denoising, the obtained clean data were integrated again to generate feature tables and representative sequences. Operational taxonomic units (OTUs) with relative abundance means of less than 0.01% were discarded, and the remaining sequences constituted the final representative sequences. Microbiome analysis and clustering of the final representative sequences were conducted using Quantitative Insights Into Microbial Ecology (QIIME1), and comparisons were made with the Greengenes database (version 13.8). We annotated the OTUs with species classification information using the Ribosomal Database Project (RDP) Classifier, counted the relative abundance of species separately, from phylum to genus, and plotted the species relative abundance distribution. The OTUs table generated was utilized for subsequent analyses.

### 2.3 Data analysis

#### 2.3.1 Confounder analysis

Analysis of variance (ANOVA)-type analyses quantified the influence of potential confounders and disease status associated with GC on individual genera (Wirbel et al., 2019). The total variance in a given genus abundance was compared to that explained by disease status and confounders (age, body mass index (BMI), *HP*, biopsy site, sex, and study), similar to a linear model. Given the non-Gaussian distribution of microbiota abundance, variance calculations were performed on rankings. Continuous values of potential confounders were converted to discrete variables, either to quartiles or by categorizing individuals according to BMI as (thin < 25, 25 < obese < 30, and overweight > 30) (Wang et al., 2022).

#### 2.3.2 Microbial diversity analysis

Microbial diversity analysis was conducted using the vegan package in R. The Shannon and Chao 1 indices estimated the microbial alpha diversity based on the OTUs table, and the Wilcoxon Rank-Sum test was employed to compare differences between the two groups. The Beta diversity of the microbiota between the samples was measured using the phyloseq package in R, according to the Bray-Curtis difference matrix, and visualized with principal coordinate analysis (PCoA). Additionally, analysis of similarities (Anosim) was applied to assess the significance of differences between different groups.

#### 2.3.3 Species difference analysis

Based on the feature table and species annotation results, linear discriminant analysis (LDA) effect size (LEfSe) (LDA > 2.0, p < 0.05) was utilized to identify biomarkers between groups, which are species or genes with significant differences between groups (Chang et al., 2022). The Kruskal-Wallis Rank-Sum test was employed for LEfSe analysis.

#### 2.3.4 Microbial correlation network analysis

Spearman correlation coefficients were used to assess the correlations between differential genera identified from the LEfSe analysis using the R package cooccur (Zou et al., 2017). Visualization was performed using Cytoscape V.3.7.2, which displayed significant co-occurrence and co-exclusion interactions (correlation coefficients ≥ 0.3, p < 0.01).

#### 2.3.5 Function prediction

Functional prediction was conducted using Phylogenetic Investigation of Communities by Reconstruction of Unobserved States (PICRUSt) (v2.4.2) software (Langille et al., 2013). The Kyoto Encyclopedia of Genes and Genomes (KEGG) database was used for functional annotation, and functional abundance profiles were obtained (Kanehisa et al., 2021). The pathways that were significantly altered between the two groups were identified using LEfSe (Logarithm value > 3.0, p < 0.01) at the KEGG level 3.

#### 2.3.6 Construction and validation of the machine learning models

The random forest (RF) package in R was utilized for modeling, employing default parameters to differentiate between GC and healthy individuals. Based on species annotation results, the relative abundance dataset was randomized into training and test sets, which were separately trained and validated for performance in a 7:3 ratio at the genus level (Liu et al., 2022b). “Mean decrease accuracy” was employed as a screening metric to identify core biomarkers for modeling. Subsequently, a ten-fold cross-validation of the RF model was conducted to determine the model error values. The diagnostic capability of the model was evaluated by plotting the receiver operating characteristic (ROC) curve using the R package pROC and calculating the area under the curve (AUC) (Han et al., 2023). External validation was also performed, incorporating additional independent data to confirm the model’s reproducibility. Furthermore, study-to-study transfer and leave-one-dataset-out (LODO) validations were conducted to demonstrate the model’s generalizability. In study-to-study transfer validation, one study was used to construct the RF model, and the remaining studies served as external test data to evaluate the model. In LODO validation, data from one study was used as the test set, while the remaining data served as the training set. AUC crossover calculations were then performed on the identified important features, and heat maps were plotted (Wirbel et al., 2019).

## 3 Results

### 3.1 Characteristics of the data sets in meta-analysis

**Figure 1:**
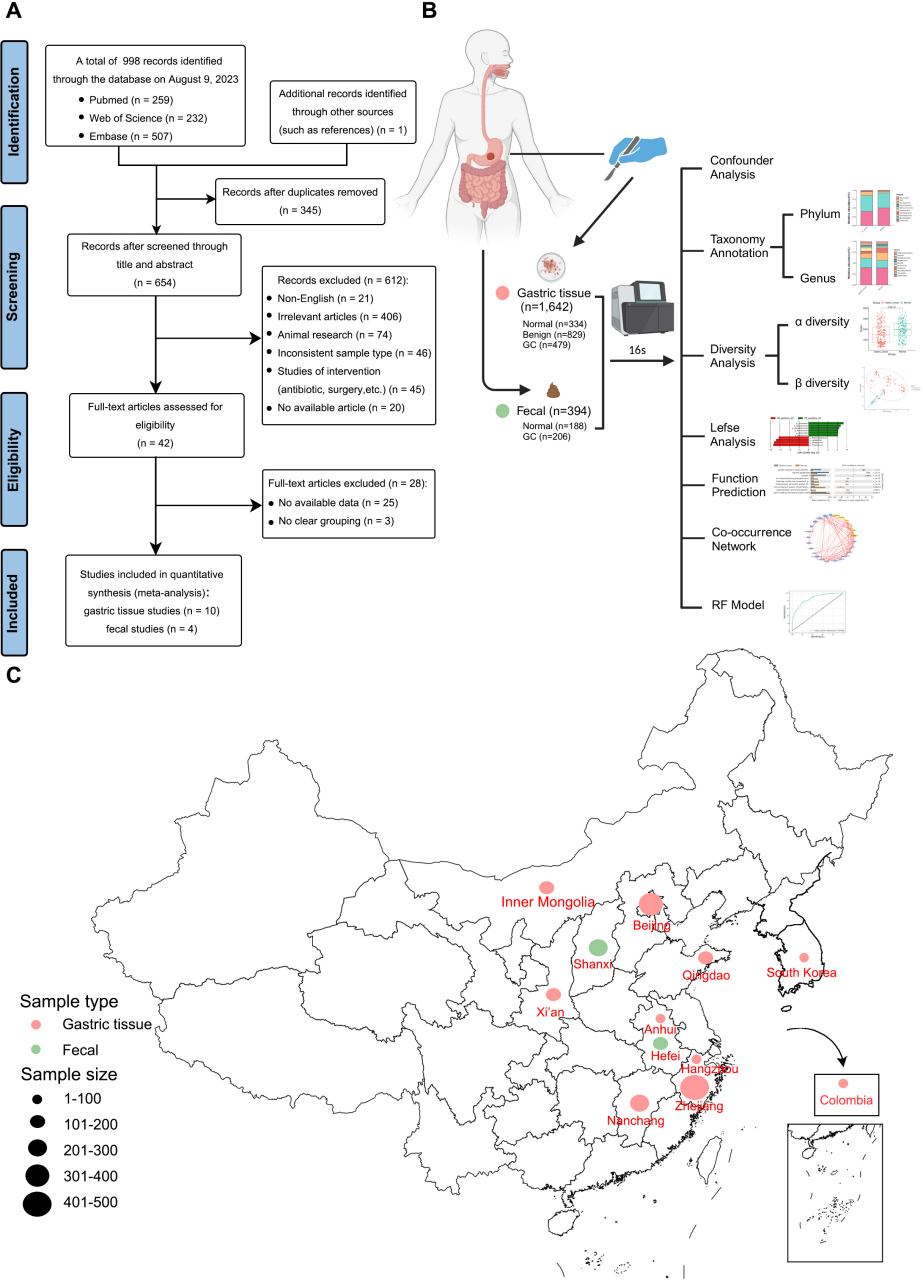
Meta-analysis flowchart. **(A)** Flowchart of the screening article process. **(B)** Schematic diagram of sample collection and bioinformatics workflow (Created with BioRender.com). **(C)** Geographic location of the dataset used in this study, with larger points representing larger sample sizes.

According to the inclusion criteria, 998 articles retrieved from PubMed, Web of Science, and Embase databases were critically reviewed, and one additional record was identified by reviewing references in the included literature. **Figure 1A** illustrates the screening process for this study. A total of 14 studies met our inclusion criteria. One study was excluded because species information could not be annotated during data processing (Ferreira et al., 2018). Ultimately, 16S rRNA sequencing datasets from 11 studies were included to estimate the signatures of gastrointestinal microbial communities associated with gastric carcinogenesis. Two additional studies were used to validate classification models. Ethical approval and written informed consent from patients were obtained for all included studies. Details of all cohorts used in this meta-analysis are provided in **Table 1**, which included 394 stool samples and 1,642 gastric tissue samples. Sample collection methods (**Figure 1B**) and processing methods for each study are detailed in **Supplementary Table S2** and **Supplementary Table S3**. These studies involved populations from China, South Korea, and Colombia, with a majority focused on Asia, particularly China (**Figure 1C**).

**Table 1:** Study characteristics of gastric tissue and intestinal datasets included in the meta-analysis.

No.	Authors/year	Bioproject	Sample Type	Country/Region	Sequencing region	Sequencing platform	Study Group
1	He, 2022 (He et al., 2022a)	PRJNA481413	Gastric tissue	China, Nanchang	V4	Illumina MiSeq	Normal (28) Benign (204) GC (57)
2	Liu D, 2021 (Liu et al., 2021a)	PRJNA678413	Gastric tissue	China, Anhui	V3-V4	Illumina NovaSeq PE250	Benign (63)
3	Wang Z, 2020 (Wang et al., 2020b)	PRJEB26931	Gastric tissue	China, Beijing	V4	Illumina MiSeq PE300	Normal (56) Benign (170) GC (84)
4	Wang L, 2020 (Wang et al., 2020a)	PRJNA313391	Gastric tissue	China, Qingdao	V3-V4	Illumina HiSeq 2500	Benign (60) GC (60)
5	Liu X, 2019 (Liu et al., 2019)	PRJNA428883	Gastric tissue	China, Zhejiang	V3-V4	Illumina MiSeq	Normal (250) GC (229)
6	Coker, 2018 (Coker et al., 2018)	PRJNA375772	Gastric tissue	China, Xi’an	V4	Illumina MiSeq	Benign (165) GC (19)
				China, Inner Mongolia	V4	Illumina MiSeq	Benign (107) GC (19)
7	Yang, 2016 (Yang et al., 2016)	PRJEB11763	Gastric tissue	Colombia	V1-V3	454 GS FLX	Benign (40)
8	Eun, 2014 (Eun et al., 2014)	PRJNA239281	Gastric tissue	South Korea, Hanyang	V5	454 GS FLX	Benign (20) GC (11)
9	Chen C, 2022 (Chen et al., 2022)	PRJNA817689	Fecal	China, Hangzhou	V4	Illumina Novaseq 6000	Normal (30) GC (41)
10	Zhang C, 2022 (Zhang et al., 2022a)	PRJNA778008	Fecal	China, Hefei	V4	Illumina Novaseq	Normal (70) GC (49)
11	Qi, 2019 (Qi et al., 2019)	PRJNA478252	Fecal	China, Shanxi	V3-V4	Illumina MiSeq	Normal (88) GC (116)
Independent validation 1	Ling, 2019 (Ling et al., 2019)	PRJNA508819	Gastric tissue	China, Zhejiang	V3-V4	Illumina MiSeq	Normal (60) GC (59)
Independent validation 2	Zhang Y, 2021 (Zhang et al., 2021)	PRJNA639644	Fecal	China, Zhejiang	V4	Illumina HiSeq 4000	Normal (39) GC (33)

GC, gastric cancer.

### 3.2 Confounder analysis of microbiota associated with GC

Considering the biological and technical variations among studies, we quantified the impact of all possible confounders associated with GC (age, BMI, *HP*, biopsy site, sex, and study) on gastrointestinal microbiota composition and compared these with disease status. The results indicated that the study, biopsy site, and *HP* status exerted the most significant impacts on microbiota composition (**Supplementary Figures S1–S3**).

### 3.3 Alterations in the composition of the gastric microbiota and its functions and networks in GC

#### 3.3.1 Alterations in the composition of the gastric microbiota in GC

**Figure 2:**
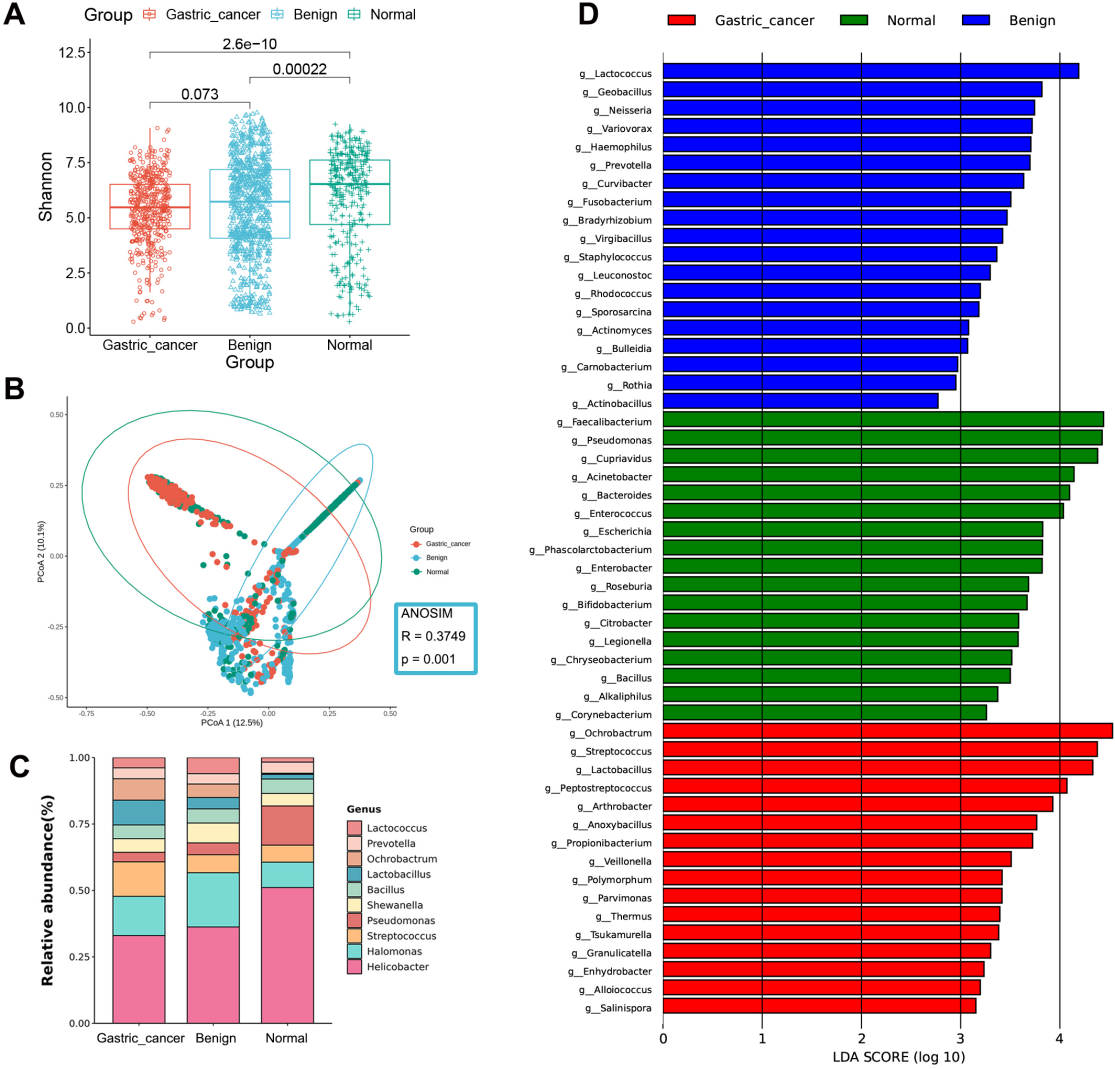
Gastric microbial composition and differential analysis of gastric cancer, benign, and normal groups. **(A)** Comparison of α-diversity among the three groups using the Shannon index. **(B)** PCoA plots based on the Bray-Curtis distance show differences in β-diversity between samples. **(C)** Taxonomic composition of gastric tissue samples at the genus level. **(D)** Bar chart of the distribution of LDA values (LDA > 2). PCoA, principal coordinate analysis; LDA, linear discriminant analysis.

The Shannon index revealed the highest microbial diversity was in the normal group compared to other stages. No significant difference was found between the benign and GC groups (**Figure 2A**). The Chao1 index (**Supplementary Figure S4A**) indicated the highest abundance in the benign group, followed by the normal group, with the lowest in the GC group, likely influenced by the larger sample size of the benign group. PCoA demonstrated that the diversity captured by the first two principal coordinates accounted for about 23% (**Figure 2B**). Due to the large size of the gastric tissue samples, some samples appeared to overlap. Still, the ANOSIM (R = 0.3749, P = 0.001) confirmed significant differences between the three stages, indicating that the samples could still be separated.

At the phylum level, the gastric microbiota was dominated by Proteobacteria, Firmicutes, Bacteroidetes, Actinobacteria, and Cyanobacteria (**Supplementary Figure S5A**). Firmicutes and Actinobacteria increased sequentially over the course of the disease, whereas Bacteroidetes decreased. At the genus level, the abundance of *Streptococcus*, *Lactobacillus*, and *Ochrobactrum* was significantly higher in the GC stage compared to the non-cancerous stages, while *Helicobacter* and *Pseudomonas* were significantly less abundant (**Figure 2C**).

LEfSe analysis was employed to identify differences in bacterial taxa between the GC, benign, and normal groups. The GC group was characterized by a higher presence of *Ochrobactrum* and *Streptococcus*, the benign group by *Lactococcus* and *Geobacillus*, and the normal group by *Faecalibacterium* and *Pseudomonas* (**Figure 2D**, **Supplementary Figure S6A** and **Supplementary Table S4**).

#### 3.3.2 Association network analysis of the gastric microbiota

**Figure 3:**
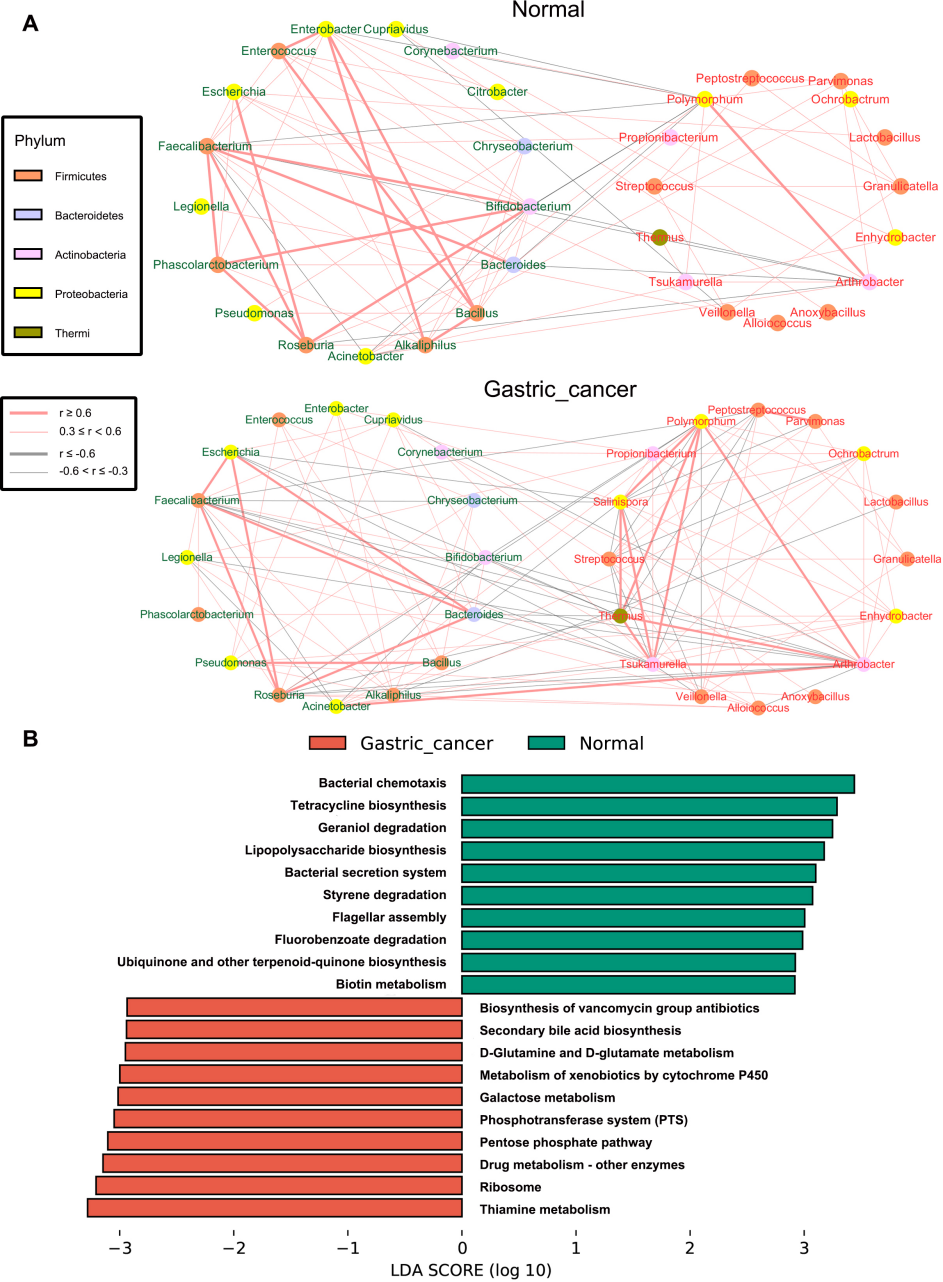
Correlation network analysis and differential functional prediction of gastric microbiota. **(A)** Correlation network of gastric cancer-associated differential bacteria in normal and gastric cancer groups. The Spearman algorithm estimated correlation strengths (|ρ| ≥ 0.3, p < 0.01). The left circle shows gastric cancer-associated depleted bacteria and the right circle shows gastric cancer-associated enriched bacteria. The depleted bacteria are marked in green font, while the enriched bacteria are marked in red font. Different colored dots indicate different phylum levels. Red lines indicate co-occurrence, gray lines indicate co-exclusion and the thickness of the line indicates the strength of the interaction. **(B)** The differential functions between normal and gastric cancer groups were predicted. The LDA bar plot displays the top 20 pathways that are significantly altered (Logarithm value > 3.0, P < 0.01). LDA, linear discriminant analysis.

To examine the interactions between GC-enriched and GC-depleted bacteria from the LEfSe analysis, we constructed association networks of the gastric microbiota by calculating correlations in the normal and GC groups using the Spearman correlation coefficient. As illustrated in **Figure 3A**, interactions within the gastric microbiota primarily occurred between Firmicutes and Proteobacteria, the two dominant phyla. The networks in the GC group were more tightly clustered and exhibited more complex co-occurring interactions compared to the normal group, indicating that the development of GC may enhance pre-existing interactions within the gastric microbiota. Positive correlations between GC-enriched bacteria, such as those between *Polymorphum* and *Arthrobacter*, gradually increased with GC progression. Conversely, positive correlations between GC-depleted bacteria decreased over the course of GC development, notably between *Bifidobacterium* and *Phascolarctobacterium*. Meanwhile, negative correlations between GC-enriched and GC-depleted bacteria gradually increased with the development of GC, such as the interactions between *Arthrobacter* and *Bifidobacterium*, *Thermus* and *Faecalibacterium*.

#### 3.3.3 Microbial functional changes in the GC

The functional potential of the gastric microbiota in the GC and normal groups was predicted using PICRUSt. Lefse analysis identified a total of 75 differential pathways between the two groups, with 35 KEGG pathways significantly up-regulated and 40 significantly down-regulated in GC (**Figure 3B**, **Supplementary Table S5**). Specifically, pathways related to carbohydrate metabolism, nucleotide metabolism, and DNA damage repair were enriched in the GC group compared to the normal group. Conversely, pathways related to biosynthesis of unsaturated fatty acids, epithelial cell signaling in *HP* infection, and bacterial synthesis and motility were reduced in GC.

### 3.4 Changes in the microbiota associated with the development of *HP*-negative and *HP*-positive GC

All gastric tissue studies in this meta-analysis declared in their methods section that *HP* testing was performed (**Supplementary Table S2**). However, many of them did not have explicit supplementary tables or information about *HP* status, which made it difficult for us to conduct discussions between *HP-*positive and *HP-*negative groups. Therefore, we attempted to take the approach of Kim et al. who determined that 1% could be used as a cutoff value for *HP* colonization by 16S rRNA gene pyrosequencing (Kim et al., 2015). Finally, we categorized samples with *HP* relative abundance greater than 1% as the *HP*-positive group and those with less than 1% as the *HP*-negative group (**Supplementary Table S6**).

**Figure 4:**
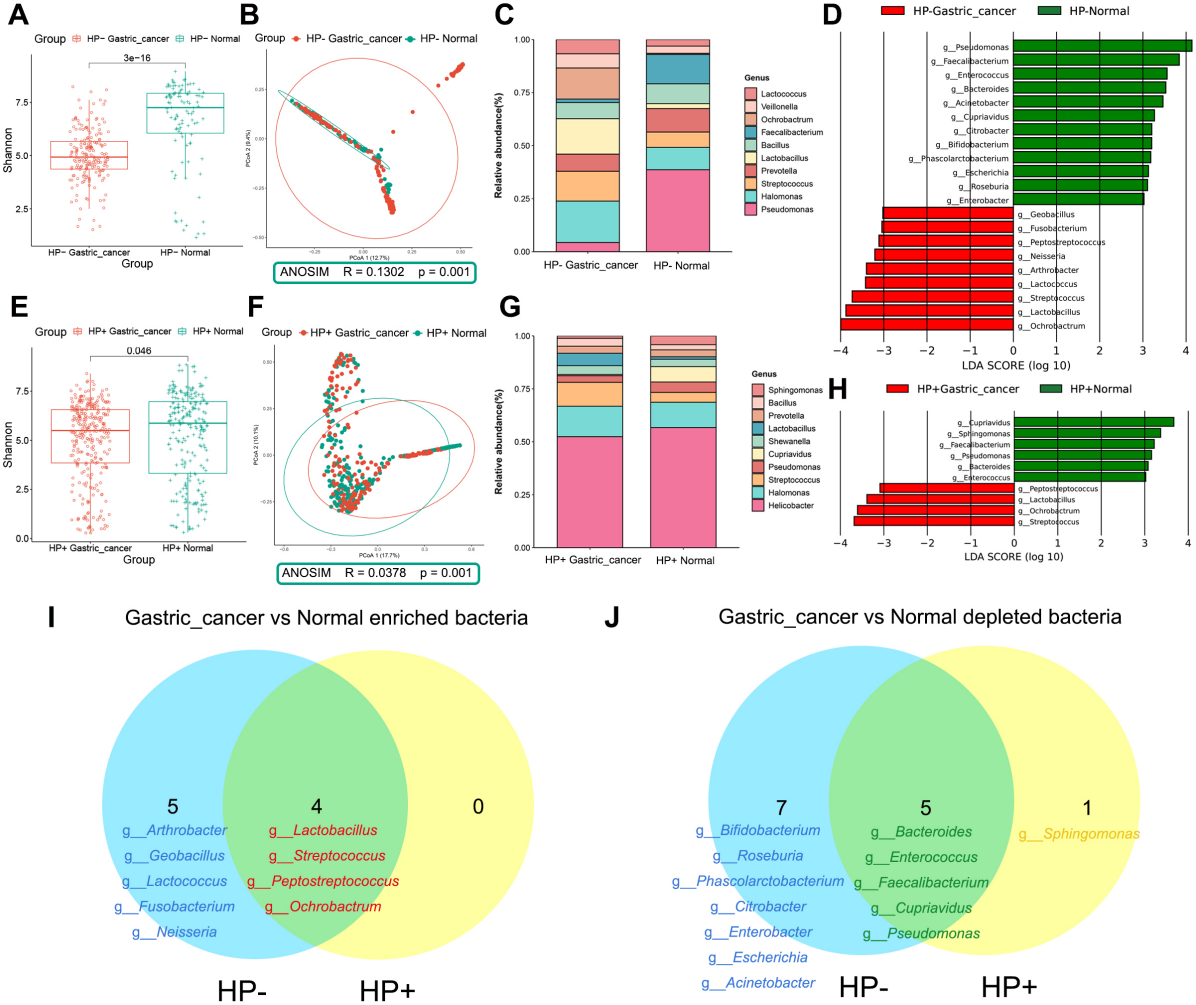
Composition and differential analysis of the gastric microbiota in the *HP*-/*HP*+ gastric cancer and normal groups. **(A)** Comparison of α-diversity between *HP*-negative normal and *HP*-negative gastric cancer groups using the Shannon index. **(B)** β-diversity was evaluated by PCoA based on Bray Curtis distance, indicating differences in composition between *HP*-negative normal and *HP*-negative gastric cancer groups. **(C)** Taxonomic composition of genus level of *HP*-negative samples. **(D)** LEfSe discriminating taxa (LDA > 3) of *HP*-negative samples. **(E)** Comparison of α-diversity between *HP*-positive normal and *HP*-positive gastric cancer groups using the Shannon index. **(F)** β-diversity was evaluated by PCoA based on Bray Curtis distance, indicating differences in composition between *HP*-positive normal and *HP*-positive gastric cancer groups. **(G)** Taxonomic composition of *HP*-positive samples at the genus level. **(H)** LEfSe discriminating taxa of *HP*-positive samples (LDA > 3). Venn diagram shows the intersection of **(I)** gastric cancer-associated enriched and **(J)** gastric cancer-associated depleted bacteria in *HP*-negative and *HP*-positive groups. *HP*-, *Helicobacter pylori*-negative; *HP*+, *Helicobacter pylori*-positive; PCoA, principal coordinate analysis; LDA, linear discriminant analysis; LEfSe, linear discriminant analysis effect size.

In *HP*-negative samples, the abundance and diversity of the gastric microbiota were significantly higher in the normal group than in the GC group, as determined by the Chao 1 index and the Shannon index (**Supplementary Figure S4C**, **Figure 4A**). In addition, based on the PCoA analysis of the Bray-Curtis distance, we found a significant difference in the distribution of the gastric microbiota between these two stages, with a distribution change of 12.7% for PCoA1 and 9.4% for PCoA2 (**Figure 4B**). Anosim further supported this conclusion (R = 0.1302, P = 0.001). The above results indicated significant differences in species diversity and composition of gastric microbiota between the *HP*-negative GC group and the *HP*-negative normal group. Proteobacteria, Firmicutes and Bacteroidetes were the dominant phyla in both groups (**Supplementary Figure S5C**). At the genus level, there was a significant increase in the abundance of *Lactobacillus*, *Streptococcus*, and *Ochrobactrum* and a decrease in the abundance of *Pseudomonas* and *Faecalibacterium* (**Figure 4C**). Twenty-one genera were identified by LEfSe analysis (**Figure 4D**, **Supplementary Table S7**), with nine genera enriched in the *HP*-negative GC group, including *Arthrobacter*, *Geobacillus*, *Lactobacillus*, *Lactococcus*, *Streptococcus*, and *Peptostreptococcus*. Conversely, twelve genera were depleted in this group, including *Bifidobacterium*, *Bacteroides*, *Enterococcus*, *Roseburia*, *Faecalibacterium*, and *Phascolarctobacterium*.

In *HP*-positive samples, the abundance and diversity of the gastric microbiota were also higher in the normal group than in the GC group (**Supplementary Figure S4D**, **Figure 4E**). The PCoA result showed a significant separation between the *HP*-positive normal group and the *HP*-positive GC group (**Figure 4F**, Anosim, R = 0.0378, P = 0.001). Proteobacteria was predominant at the phylum level, especially in the *HP*-positive normal group (**Supplementary Figure S5D**). At the genus level, changes in *Lactobacillus* and *Streptococcus* in the *HP*-positive normal and GC groups paralleled those in the *HP*-negative group (**Figure 4G**). LEfSe analysis indicated that *Lactobacillus*, *Streptococcus*, *Peptostreptococcus*, and *Ochrobactrum* were enriched, whereas *Bacteroides*, *Enterococcus*, *Faecalibacterium*, *Sphingomonas*, *Cupriavidus*, and *Pseudomonas* were depleted in the *HP*-positive GC group compared to the *HP*-positive normal group (**Figure 4H**, **Supplementary Table S8**).

Finally, the Venn diagram revealed that *Lactobacillus*, *Streptococcus*, *Peptostreptococcus*, and *Ochrobactrum* were enriched, whereas *Bacteroides*, *Enterococcus*, *Faecalibacterium*, *Cupriavidus*, and *Pseudomonas* were depleted in both *HP*-negative and *HP*-positive GCs. (**Figures 4I, J**). However, genera such as *Arthrobacter*, *Geobacillus*, *Lactococcus*, *Fusobacterium*, and *Neisseria* only promote the development of *HP*-negative GC.

### 3.5 Alterations in the composition of the gut microbiota in GC

**Figure 5:**
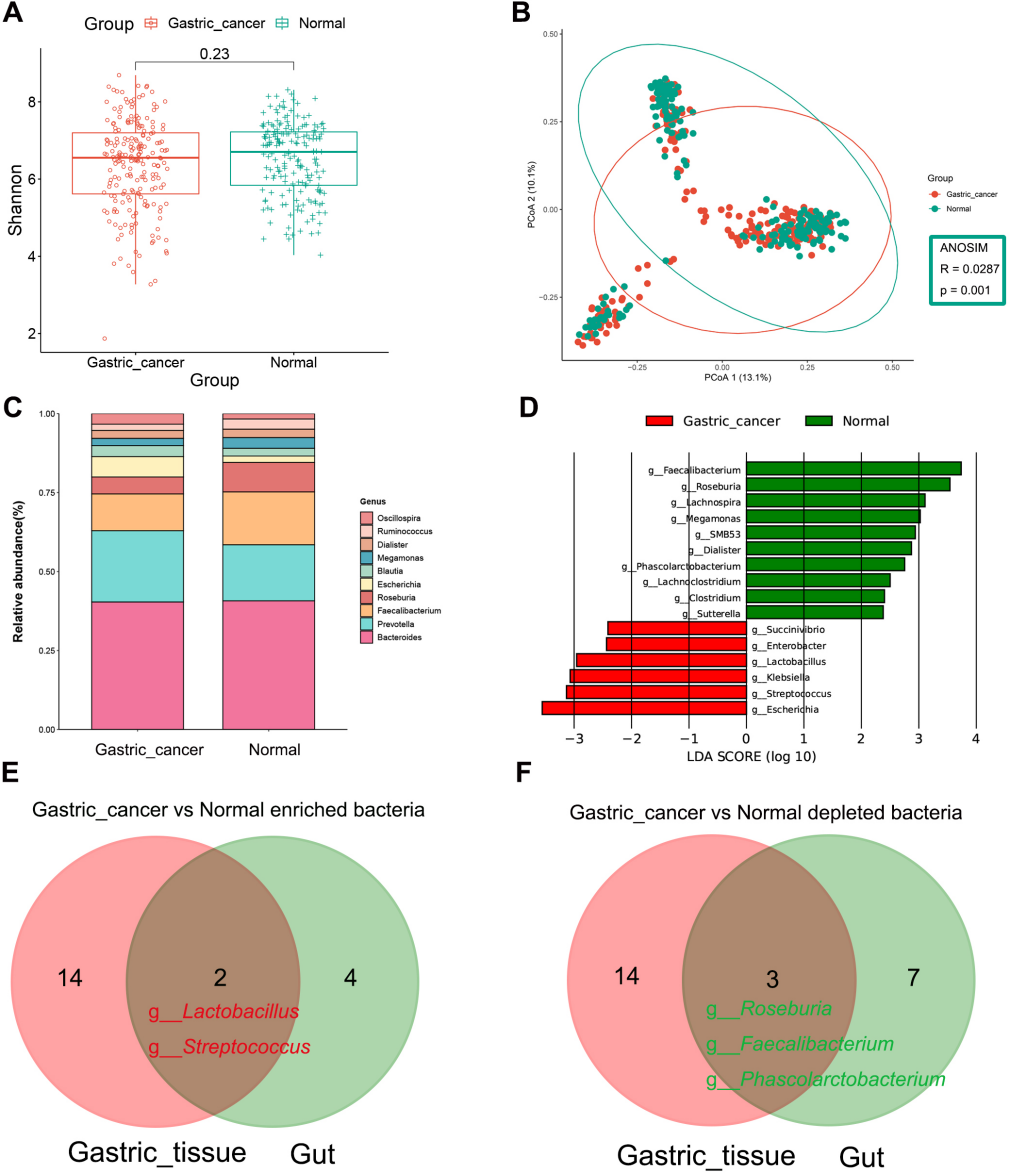
Composition and differential analysis of the gut microbiota in gastric cancer patients and healthy individuals. **(A)** α-diversity was compared between gastric cancer and normal groups using the Shannon index. **(B)** β-diversity was evaluated by PCoA based on Bray Curtis distance, indicating differences in composition between groups. **(C)** Genus-level classification profiles of gastric cancer patients and healthy individuals. **(D)** Bar chart of the distribution of LDA values (LDA > 2). Venn diagram shows the intersection of **(E)** gastric cancer-associated enriched and **(F)** gastric cancer-associated depleted bacteria in gastric tissues and gut. PCoA, principal coordinate analysis; LDA, linear discriminant analysis.

The abundance of gut microbiota was higher in healthy individuals compared to those in GC patients (**Supplementary Figure S4B**) However, the two groups did not differ significantly in α-diversity (**Figure 5A**), aligning with previous findings (Liu et al., 2021b; He et al., 2022b; Li et al., 2022; Chen et al., 2022). However, a notable difference in β-diversity between the two groups was observed (**Figure 5B**, ANOSIM, R = 0.0287, P = 0.001). Next, the microbial composition of the fecal samples was synthesized at the phylum and genus levels. Bacteroidetes, Firmicutes and Proteobacteria dominated the intestinal microbiota as phylum, which accounted for more than 95% of the sequence (**Supplementary Figure S5B**). *Prevotella* and *Escherichia* were enriched in GC at the genus level compared to the normal group, while the abundance of *Faecalibacterium* and *Roseburia* was significantly reduced in GC (**Figure 5C**). LEfSe difference comparisons identified *Lactobacillus*, *Streptococcus*, *Succinivibrio*, *Enterobacter*, *Escherichia* and *Klebsiella* as enriched in the GC group compared to the normal group. Meanwhile, there were ten genera reduced in GC, including *Clostridium*, *SMB53*, *Lachnoclostridium*, *Lachnospira*, *Roseburia* and *Faecalibacterium* (**Figure 5D**, **Supplementary Figure S6B** and **Supplementary Table S9**).

Venn diagrams highlighted co-differentiating bacteria in the stomach and intestines, including the GC-enriched bacteria, *Lactobacillus* and *Streptococcus*, and the GC-depleted bacteria, *Roseburia*, *Faecalibacterium* and *Phascolarctobacterium* (**Figures 5E, F**).

### 3.6 Microbial markers for distinguishing GC patients from healthy individuals

**Figure 6:**
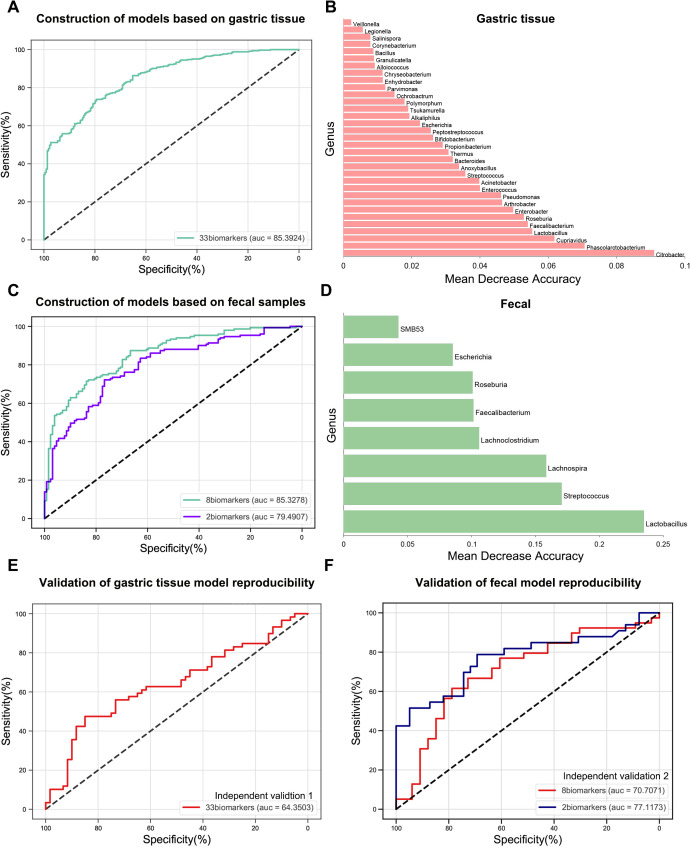
Construction and validation of gastric cancer diagnostic models based on gastric and gut-specific microbiota. **(A)** The RF model of GC versus normal groups was constructed in gastric tissue samples. **(B)** The weight shares of mean decrease accuracy for the 33 most discriminatory bacterial taxa identified in the RF model in **(A)**. **(C)** RF models for the GC versus normal groups were constructed in fecal samples. **(D)** The weight shares of mean decrease accuracy for the 8 most discriminatory bacterial taxa identified in the RF model in **(C)**. **(E)** In independent validation 1, the RF model in **(A)** was validated. **(F)** In independent validation 2, two RF models in **(C)** were validated. RF, random forest; GC, gastric cancer.

Using the results from the LEfSe analysis, we developed RF models to investigate the ability of gastrointestinal microbiota to differentiate between GC patients and healthy individuals. In the gastric tissue, the taxa that significantly changed between normal and GC groups included 16 GC-enriched genera and 17 GC-depleted genera (**Figure 2D**, **Supplementary Table S4**), which 33 genera were able to maximize the differentiation between healthy individuals and patients with GC (AUC = 0.8539, **Figures 6A, B**). In addition, in the intestine, 16 genera (six enriched in GC and ten depleted in GC) demonstrated significant changes between the normal and GC groups (**Figure 5D**, **Supplementary Table S9**). The results showed that a minimal set of 8 genera effectively distinguished healthy individuals from GC patients (AUC = 0.8533, **Figures 6C, D**). *Lactobacillus* and *Streptococcus* accounted for a significant share in this RF model. For better clinical dissemination, we reconstructed an RF model based on *Lactobacillus* and *Streptococcus* in fecal samples (AUC = 0.7949, **Figure 6C**).

### 3.7 Validation of microbial classifiers

Two additional independent cohorts from Zhejiang (Independent Validation 1 and Independent Validation 2) were included in this study to validate the RF models’ reproducibility. Independent Validation 1 comprised 60 normal and 59 GC tissue samples. Independent Validation 2 consisted of raw data from the gut microbiome of 39 healthy individuals and 33 GC patients (**Table 1**). The RF model based on gut microbiota demonstrated higher reproducibility than that of gastric microbiota (**Figures 6E, F**). Furthermore, the AUC value of the model validated with the combination of *Lactobacillus* and *Streptococcus* (AUC = 0.7712) was higher than that of eight microbial markers (AUC = 0.7071) (**Figure 6F**). These results suggest that the combination of *Lactobacillus* and *Streptococcus* offers strong discriminatory capability and high reproducibility as biomarkers.

Additionally, to mitigate the impacts of geographic and technological differences, study-to-study transfer and LODO validations were conducted for all cohorts involved in the gut study to assess the generalizability of the *Lactobacillus* and *Streptococcus* combination across multiple studies. The AUC values for the study-to-study transfer validation ranged from 0.68 to 0.89 (mean AUC = 0.79), and AUC values for LODO validation ranged from 0.76 to 0.86 (mean AUC = 0.81) (**Supplementary Figure S7**).

## 4 Discussion

In this study, we conducted a meta-analysis of multiple published 16S rRNA datasets and, for the first time, incorporated fecal samples to characterize the gastric and gut microbial communities associated with gastric carcinogenesis, as well as to evaluate the potential of microbial markers in distinguishing between GC patients and healthy individuals.

Our results showed that the abundance and diversity of the gastric microbiota are significantly reduced in GC compared to the precancerous stage, aligning with prior research (Wang et al., 2020b; Liu et al., 2022a; Sun et al., 2022). However, the changes in the α-diversity of the gastric microbiota across the GC cascade lack consistency (Eun et al., 2014; Castaño-Rodríguez et al., 2017), which could be addressed by enlarging the sample size and standardizing the metrics used for assessing diversity. Moreover, the phyla and genera dominating the gastric and gut microbiota derived from our meta-analysis also align with previous studies (Zhang et al., 2021; Liu et al., 2022a), although with minor variations in the proportions of specific phyla and genera. These differences may stem from unavoidable factors such as analytical methods, geographic location, and racial heterogeneity. LEfSe analysis identified *Lactobacillus* and *Streptococcus* as GC-enriched bacteria in both the stomach and intestines. Conversely, *Roseburia*, *Faecalibacterium* and *Phascolarctobacterium* were found to be GC-depleted bacteria in both sites. The enrichment of *Lactobacillus* and *Streptococcus* in GC has been observed in many current studies (Ferreira et al., 2018; Wang et al., 2020b), and they have also been shown to promote the development of GC through various pathways. First, *Lactobacillus* produces lactic acid, which can serve as the energy source for tumor cells and promote their proliferation (Vinasco et al., 2019). Moreover, *Lactobacillus* can upregulate inflammatory factors such as Ptger4 and Tgf-β, which promote inflammatory responses (Lertpiriyapong et al., 2014). In addition, both *Lactobacillus* and *Streptococcus* can contribute to producing N-nitroso compounds, which are highly carcinogenic (Jo et al., 2016; Li and Perez Perez, 2018). In contrast, *Roseburia*, *Faecalibacterium* and *Phascolarctobacterium*, which are beneficial bacteria, produce butyrate, acetate and propionate. These short-chain fatty acids are crucial in inhibiting the development of GC, particularly butyrate (Chattopadhyay et al., 2022). Butyrate can inhibit the Warburg effect in GC, thereby depriving tumor cells of the necessary energy for growth (Li et al., 2018). Additionally, butyrate promotes the production of Caspase 9, leading to the apoptosis of tumor cells (Zhang et al., 2022b).

Changes in the correlations between bacteria can reflect differences between the intratumor-specific microenvironment and healthy individuals, thereby improving our understanding of gastric carcinogenesis. The positive correlation between GC-enriched bacteria, which contribute to gastric carcinogenesis, increased during the GC stage, while the positive correlation between GC-depleted bacteria, crucial for maintaining the balance of the gastric microbiota, decreased compared to that in healthy individuals. Moreover, the gradual increase in negative correlations between GC-enriched and GC-depleted bacteria suggested a mutually antagonistic relationship. These findings indicate that alterations in the gastric microbial community may be associated with the development of GC.

Functional analysis sheds light on potential pathogenic mechanisms in GC, which could inform new approaches for its prevention and treatment. Carbohydrate metabolism and nucleotide metabolism pathways were significantly enriched in GC compared with the normal group, potentially providing more energy for tumor growth and promoting the division and proliferation of tumor cells (Martínez-Reyes and Chandel, 2021). Notably, the pathway of unsaturated fatty acid biosynthesis was significantly reduced in the GC group compared to the normal group. The biosynthesis of unsaturated fatty acids may be closely linked to the central mechanism of ferroptosis, namely lipid peroxidation aggregation. Lee et al. reported that an increase in the biosynthesis of unsaturated fatty acids such as arachidonic acid or adrenic acid could significantly enhance the sensitivity of GC to ferroptosis (Lee et al., 2020). Ferroptosis, a novel form of cell death tightly regulated by Fe^2+^, the System Xc-/glutathione/glutathione peroxidase 4 (System Xc-/GSH/GPX4) pathway and lipid metabolic pathways, differs from apoptosis and necrosis (Lei et al., 2022). There has been substantial evidence suggesting that abnormalities in the regulatory mechanisms of ferroptosis are closely linked to cancer development (Zhang et al., 2018; Jian et al., 2023). Induction of ferroptosis inhibits tumor cell growth and improves GC prognosis. Conversely, inhibition of ferroptosis promotes the development of GC. Thus, targeting ferroptosis may be a promising strategy for treating GC.

We examined the microbiota playing essential roles in developing *HP*-negative and *HP*-positive GCs separately and identified bacterial genera that changed in both conditions through Venn analysis. Some bacterial genera changed exclusively in either *HP*-negative or *HP*-positive GC. Characterizing the flora changes associated with developing *HP*-negative and *HP*-positive GCs is crucial for early intervention in *HP*-negative GC and late treatment of *HP*-positive GC. Current methods for early diagnosis of GC are predominantly *HP*-specific, including rapid urease tests, urea breath tests, and immunohistochemical analyses, leaving a gap in the detection of non-*HP* infections. This gap hinders timely detection in *HP*-negative GC. For *HP*-positive GC, many studies have found that *HP* colonization gradually decreases with GC progression (Ferreira et al., 2018; Hsieh et al., 2018; Liu et al., 2019). At the same time, some non-*HP* commensals, such as *Lactobacillus* and *Streptococcus*, gradually increase (Coker et al., 2018; Ferreira et al., 2018; Wang et al., 2020b), so studying these non-*HP* commensals can help us better treat advanced *HP*-positive GC.

The gastric and gut microbiota were comprehensively evaluated for their capability to detect early GC, demonstrating good predictive abilities with AUCs of 0.8539 and 0.8533, respectively. Given the ease of use, cost-effectiveness, and non-invasiveness, the gut microbiota-based model was deemed more suitable for early GC screening. By ranking the important features of the gut microbiota model, it was found that the combination of *Lactobacillus* and *Streptococcus* alone effectively differentiated between GC patients and healthy individuals (AUC = 0.7949). These two important features were validated in an independent cohort (AUC = 0.7712). Finally, through study-to-study transfer (mean AUC of 0.79) and LODO (mean AUC of 0.81) validations, it was demonstrated that the combination of *Lactobacillus* and *Streptococcus* could overcome technical and geographical differences to be generalizable across multiple populations. It has been found that fecal microbiota can be used as biomarkers for the non-invasive diagnosis of GC. A diagnostic model constructed by Qi et al. based on the combination of *Lachnospira*, *Lactobacillus*, *Streptococcus*, *Veillonella*, and *Tyzzerella_3* was able to discriminate well between GC patients and healthy individuals (AUC=0.95) (Qi et al., 2019). However, this model was confined to a single region and lacked independent validation, rendering its accuracy and reproducibility indeterminate (Qi et al., 2019). It is important to note that the structure and composition of the gut microbiota may be altered due to various factors. Previous studies have demonstrated a significant decrease in the richness and diversity of the gut microbiota following antibiotic use (Becattini et al., 2016). Conversely, oral probiotics may optimize the structure of the gut microbiota, potentially restoring its homeostasis (Wang et al., 2023). Additionally, it has been reported that intestinal diseases such as constipation, colorectal cancer, and ulcerative colitis are usually accompanied by varying degrees of intestinal microbiota dysbiosis or abnormalities, which are mainly characterized by a relative decrease in beneficial bacteria (e.g., *Lactobacillus* and *Bifidobacterium*), a relative increase in pathogenic bacteria (e.g., *Fusobacterium nucleatum* and *Escherichia coli*), and a decrease in species richness and diversity (Quaglio et al., 2022; Yang et al., 2022). However, the studies we included already excluded these potential factors when screening the study population, so there was no way to explore their impact on the model predictions further.

Most current studies on GC are monoecological, but joint diagnosis of GC through multiecology has also shown good performance. The RF model constructed by Zhang et al. combining oral and fecal microbiota had high accuracy (AUC= 0.922) in distinguishing between GC patients and healthy individuals (Zhang et al., 2022a). Our study also started from a multi-ecological perspective to identify shared microbiota, which may play a pivotal role in GC pathogenesis. Subsequently, based on these key microbiota, RF models were constructed in the stomach and the intestine, respectively. Compared to multi-ecological co-modeling, our models are more cost-effective and offer greater clinical translational potential, which can be achieved by only performing single-sample sampling.

Despite diligent efforts, this study faces several limitations and challenges. Its predominant focus on the Asian population may restrict the generalizability of our model to other regions, notably Europe and America. Additionally, the absence of accessible clinical information regarding tumor stage, histologic typing, and dietary behaviors limits a comprehensive evaluation of their potential influences. Furthermore, the utilization of our model for individual screening may be impacted by medication usage, specifically antibiotics and probiotics, as well as by an individual’s underlying health conditions, such as constipation, colorectal cancer, and ulcerative colitis. Therefore, future research should focus on increasing the sample size and geographic diversity while also considering a broader range of influencing factors to improve the applicability of our model to a more diverse population. We should also further explore the potential impact of the type and interval of antibiotic and probiotic use and intestinal comorbidities on the accuracy of our model. Moreover, our investigation of GC microbiota was limited by using 16S rRNA data, which is not as good as metagenomic data for resolution at the species level and functional prediction. Additionally, fungi and viruses are significant contributors to the pathogenesis of GC (Yarahmadi and Afkhami, 2024), yet their presence cannot be adequately assessed through 16S rRNA sequencing alone. Future studies should employ metagenomics sequencing technology, which allows for precise identification of bacterial species and concurrently captures data on fungi and viruses. Metagenomics also facilitates a thorough analysis of interactions among bacteria, fungi, and viruses, providing a more comprehensive understanding of the role of microecology in GC pathogenesis.

In summary, intratumoral and intestinal-specific co-differential *Lactobacillus* and *Streptococcus* were identified and could be used as markers for non-invasive early detection of GC with good accuracy across different populations. In cases of *HP*-positive GC, *Lactobacillus*, *Streptococcus*, *Peptostreptococcus* and *Ochrobactrum*, along with *HP*, may contribute to the development of GC. In *HP*-negative GC, *Arthrobacter*, *Geobacillus*, *Lactococcus*, and *Fusobacterium* independently contribute to the development of GC. The GC-depleted pathway involves promoting ferroptosis, and further research is needed on the interactions and potential mechanisms between intratumoral and fecal microbiota and their specific metabolites in gastric carcinogenesis.

## Data Availability

The original contributions presented in the study are included in the article/**Supplementary Material**. Further inquiries can be directed to the corresponding authors.
