# Supplementary material for: Intratumoral and fecal microbiota reveals microbial markers associated with gastric carcinogenesis
Source: Front Cell Infect Microbiol. 2024 Sep 17;14:1397466. doi: 10.3389/fcimb.2024.1397466 (PMC11442432; doi:10.3389/fcimb.2024.1397466)
Supplement: Supplementary file 1 [file DataSheet1.docx]

Supplementary Material

# Supplementary Figures and Tables

## Supplementary Figures

**
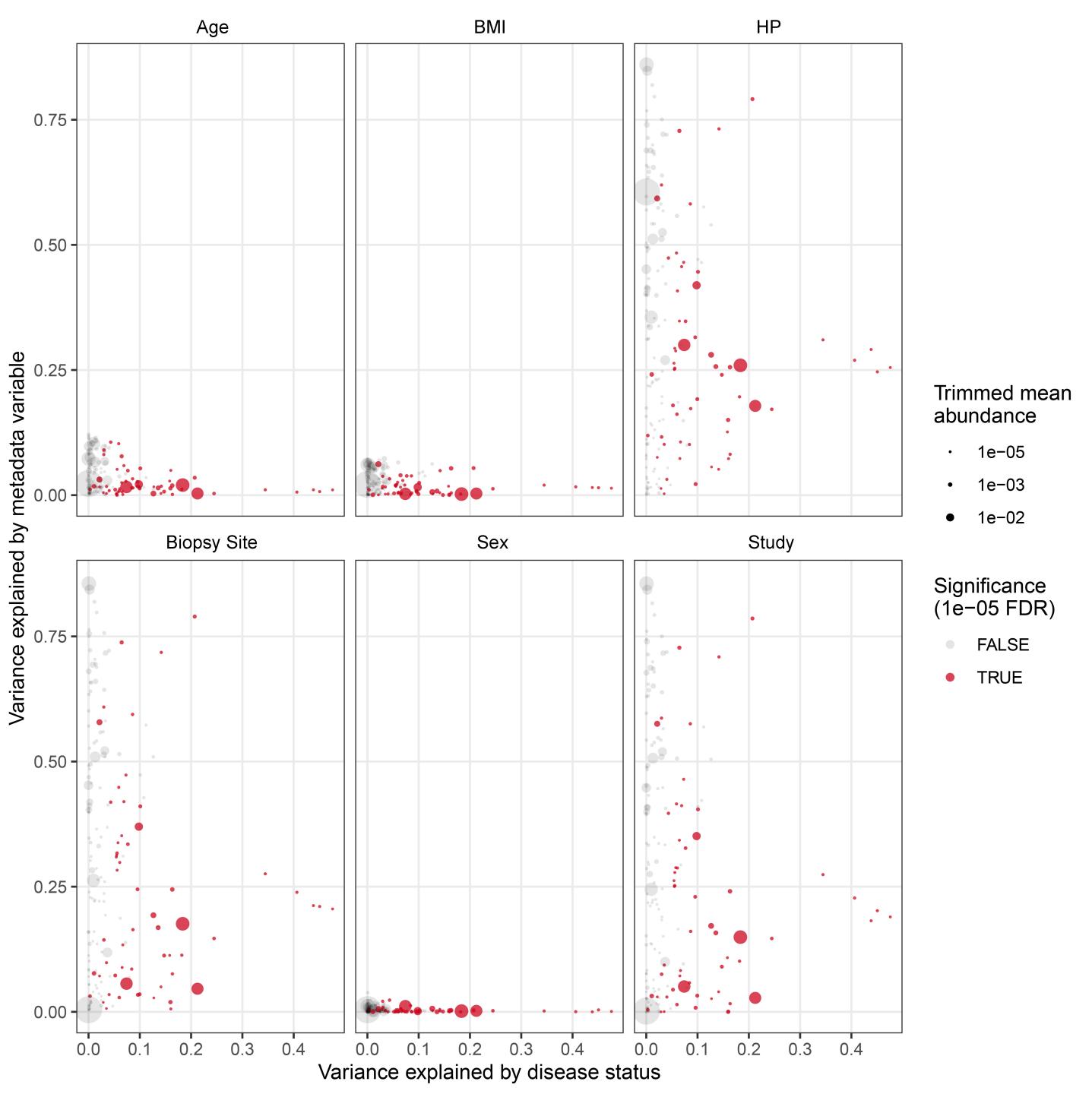
**

**Supplementary Figure 1.** In the gastric study, the variance explained by disease status (Normal versus Benign) was compared with that of individual genera explained by different potential confounders (age, BMI, *HP*, biopsy site, sex, and study). Point size is proportional to genus abundance, with the red colour indicating genera with significant differences. BMI, body mass index; *HP*, *Helicobacter pylori*.


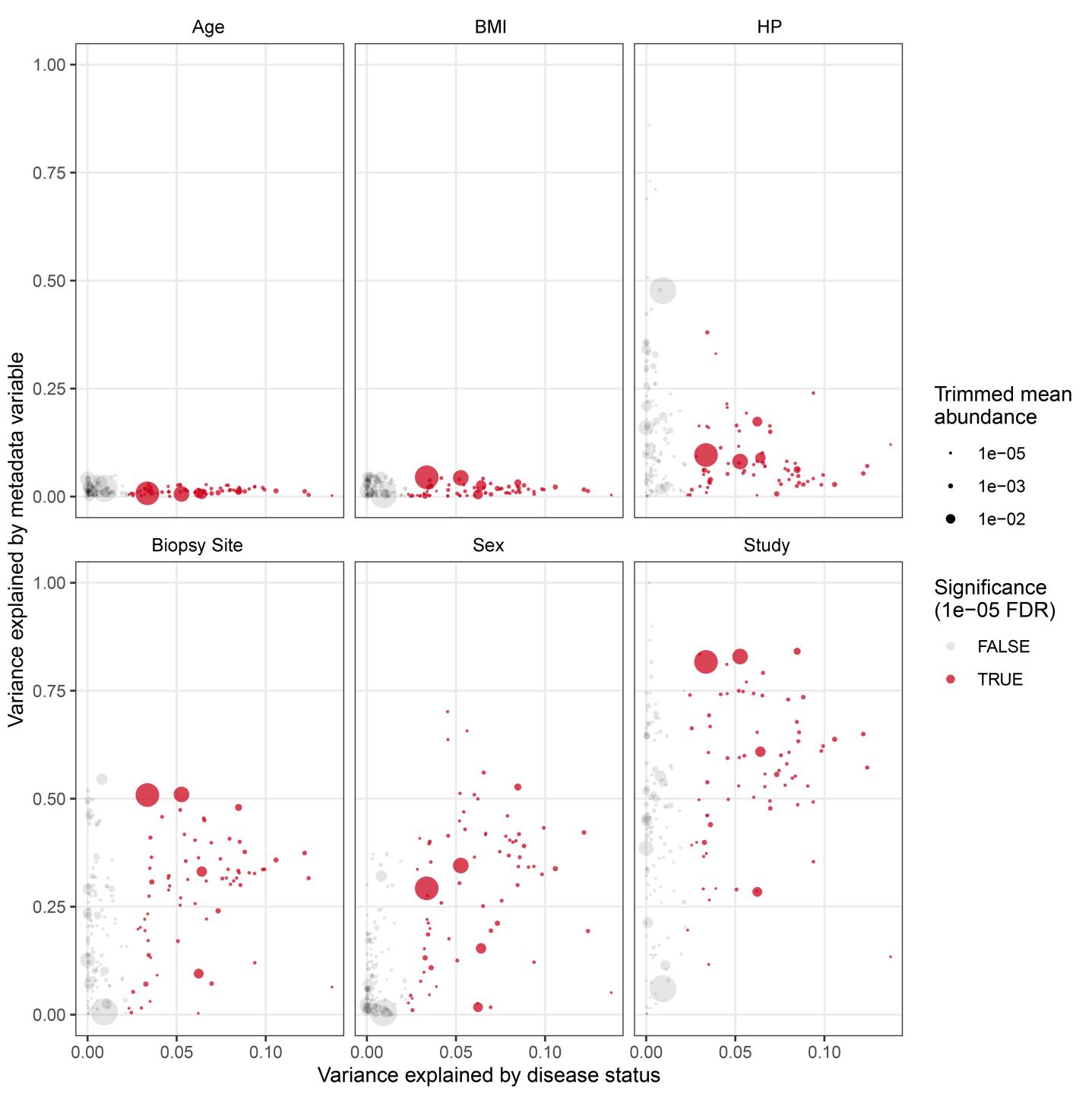


**Supplementary Figure 2.** In the gastric study, the variance explained by disease status (Benign versus Gastric cancer) was compared with that of individual genera explained by different potential confounders (age, BMI, *HP*, biopsy site, sex, and study). Point size is proportional to genus abundance, with the red colour indicating genera with significant differences. BMI, body mass index; *HP*, *Helicobacter pylori*.


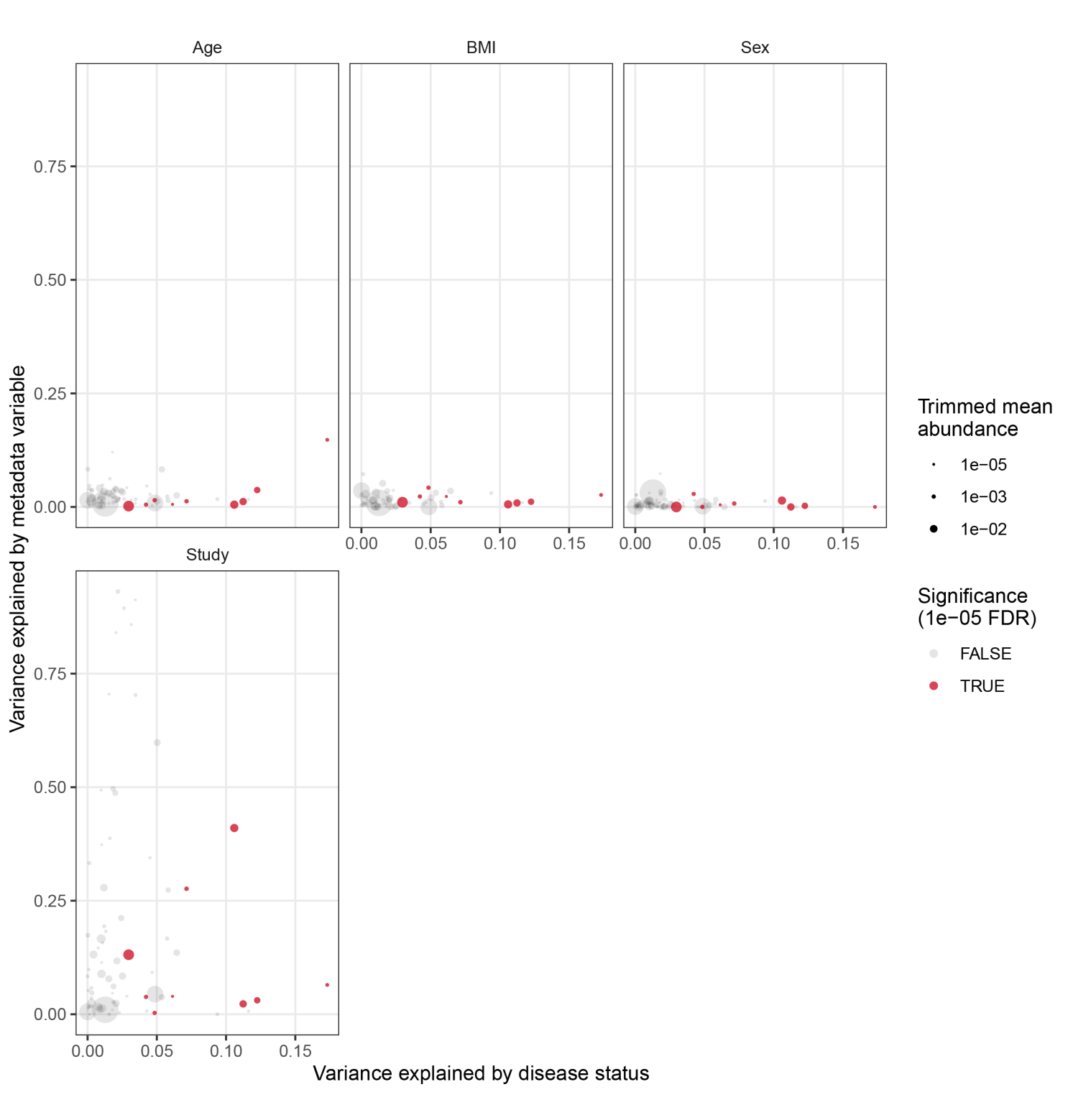


**Supplementary Figure 3.** In the gut study, the variance explained by disease status (Normal versus Gastric cancer) was compared with that of individual genera explained by different potential confounders (age, BMI, sex, and study). Point size is proportional to genus abundance, with the red colour indicating genera with significant differences. BMI, body mass index.


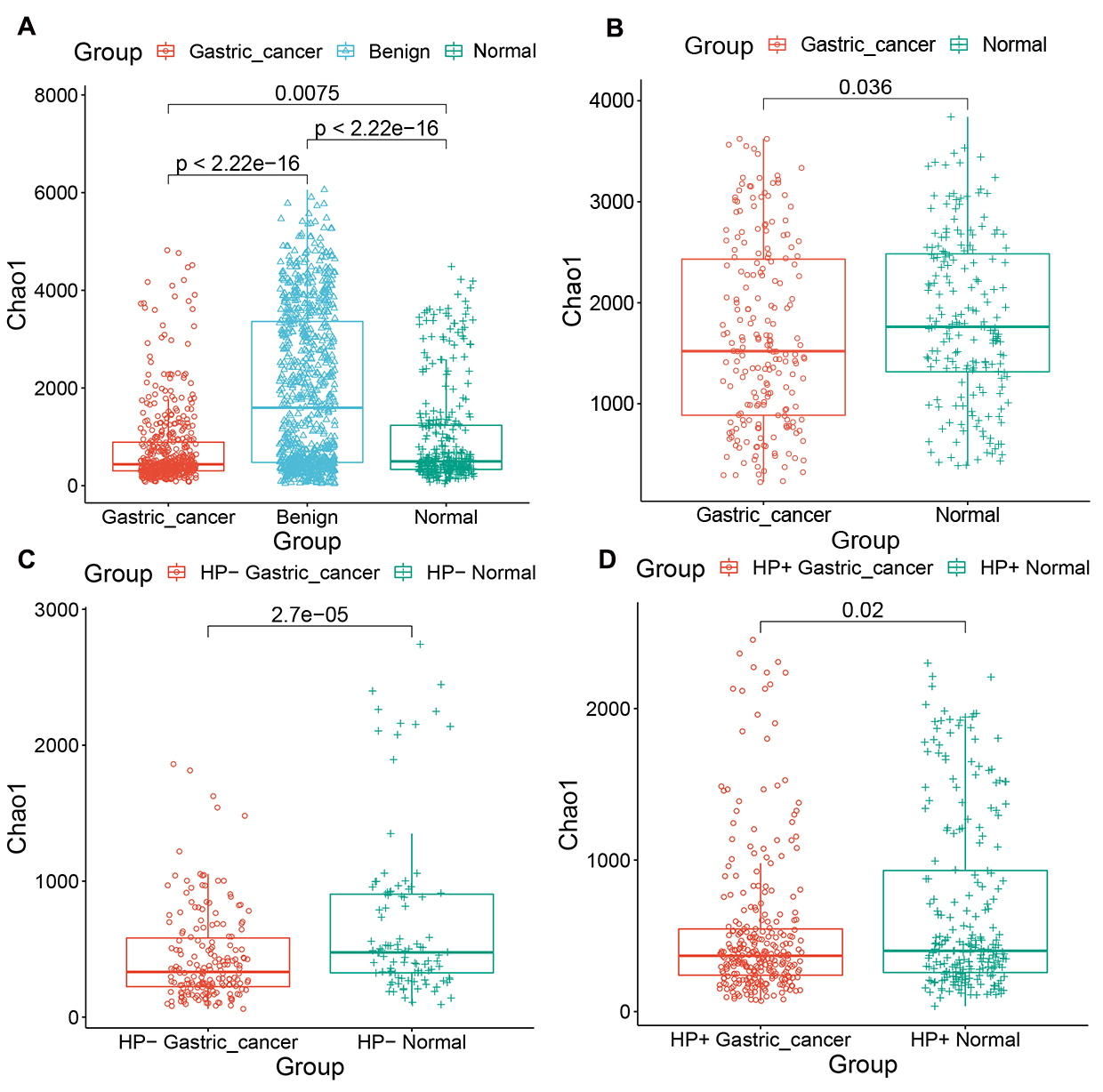


**Supplementary Figure 4.** The alpha diversity analysis shows the Chao 1 index for each group. (A) In the gastric microbiota, a comparison of the Chao1 index between the gastric cancer, benign and normal groups. (B) In the gut microbiota, a comparison of the Chao1 index between the gastric cancer and the normal groups. (C) Comparison of Chao1 index between gastric cancer and normal groups in *HP*-negative group. (D) Comparison of Chao1 index between gastric cancer and normal groups in *HP*-positive group. *HP*-, *Helicobacter pylori*-negative; *HP*+, *Helicobacter pylori*-positive.


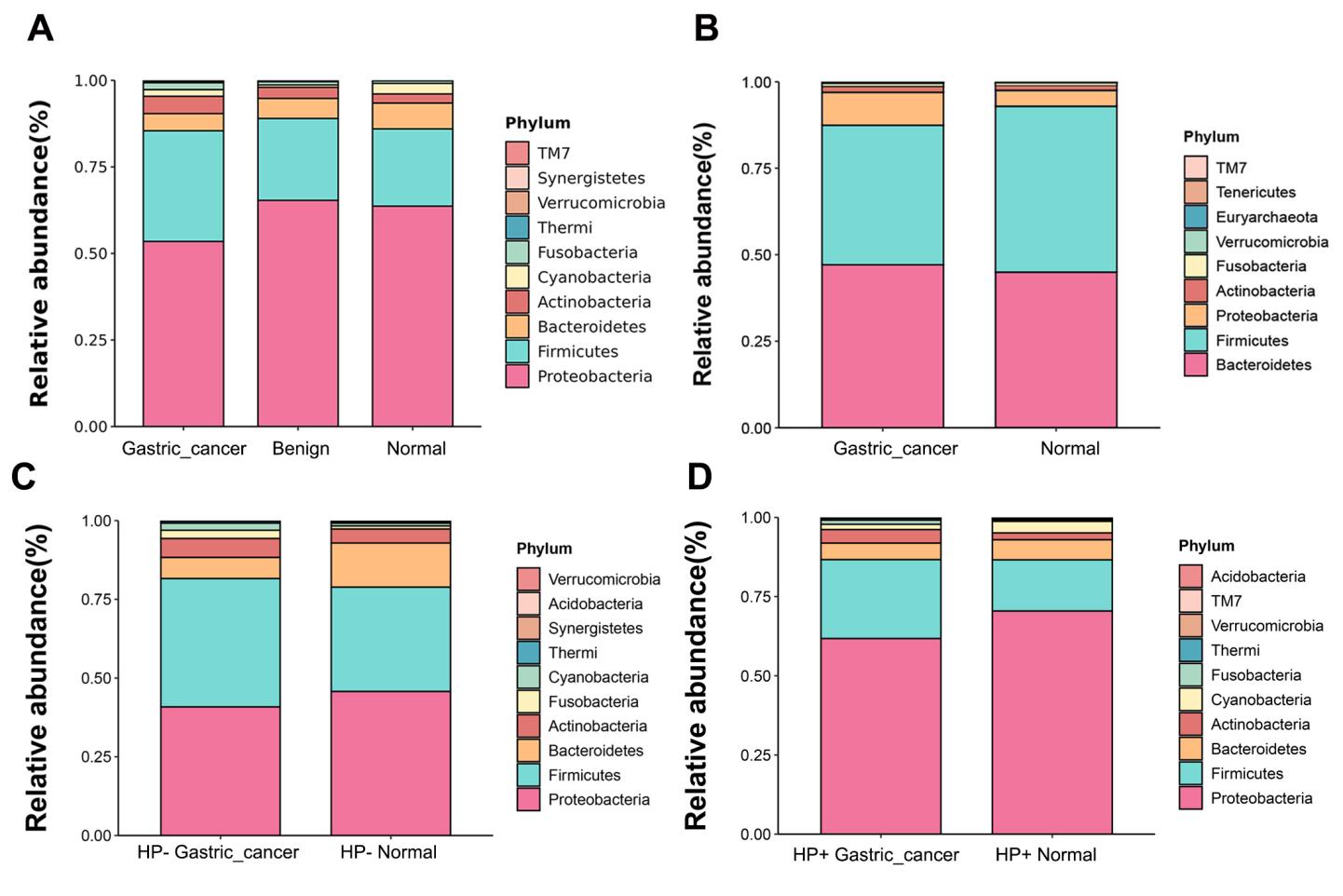


**Supplementary Figure 5.** Taxonomic composition of each group at the phylum level. (A) Taxonomic composition of the gastric cancer, benign, and normal groups at the phylum level in the gastric microbiota. (B) Taxonomic composition of gastric cancer and normal groups at the phylum level in the gut microbiota. (C) Taxonomic composition of *HP*-negative gastric cancer and *HP*-negative normal groups at the phylum level. (D) Taxonomic composition of *HP*-positive gastric cancer and *HP*-positive normal groups at the phylum level. *HP*-, *Helicobacter pylori*-negative; *HP*+, *Helicobacter pylori*-positive.


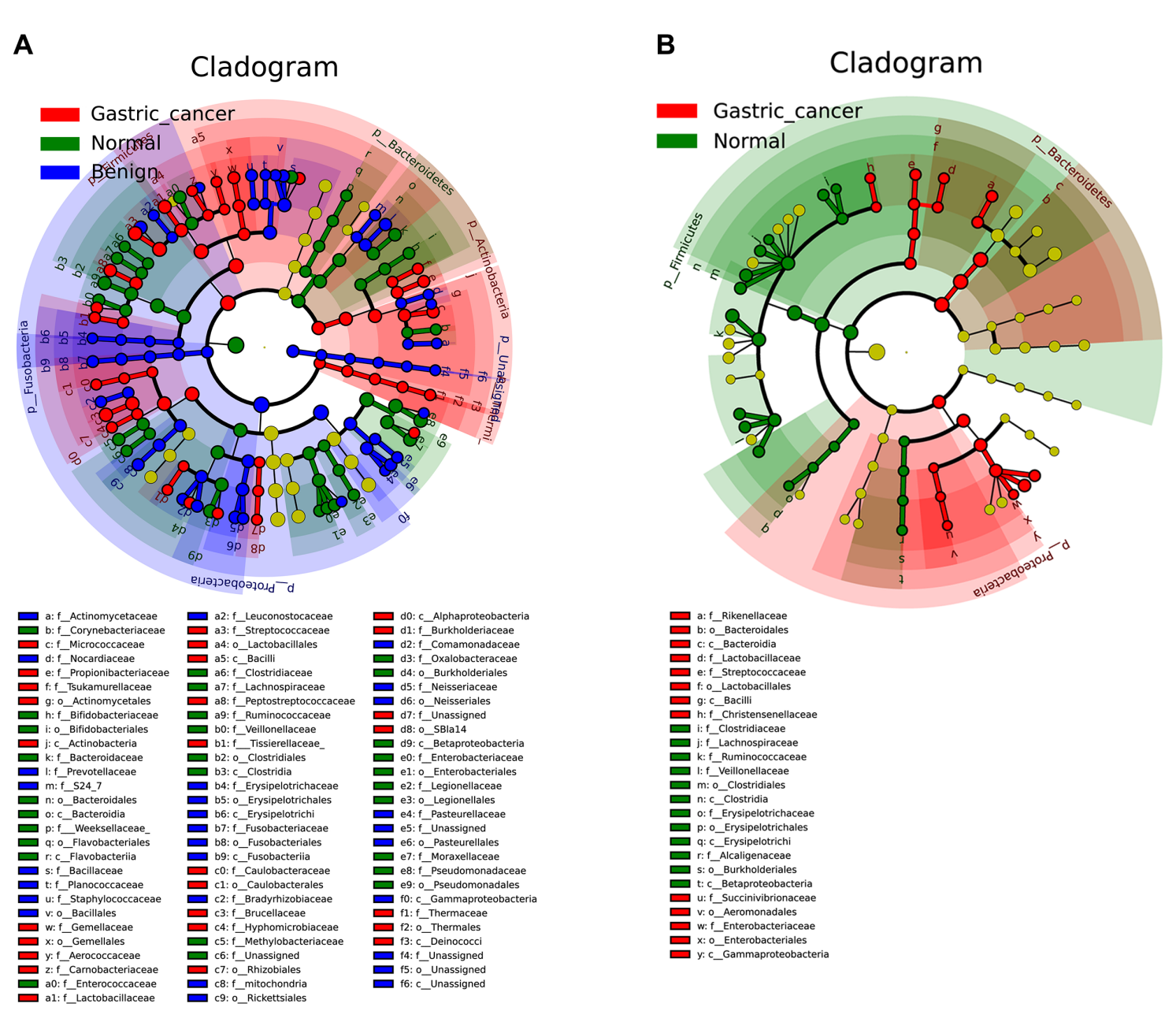


**Supplementary Figure 6.** Cladogram of Lefse analysis. (A) Gastric tissue samples. (B) Fecal samples. Only taxa with LDA > 2 are shown in the graph. Circles radiating from inside to outside represent taxonomic levels from phylum to genus. Each small circle at a different taxonomic level represents a taxon at that level, and the circle diameter is proportional to the size of the relative abundance. The yellow indicates taxa with no significant differences, the red indicates taxa enriched in the gastric cancer group, the blue indicates taxa enriched in the benign group, and the green indicates taxa enriched in the normal group. LDA, linear discriminant analysis; LEfSe, Linear discriminant analysis Effect Size.


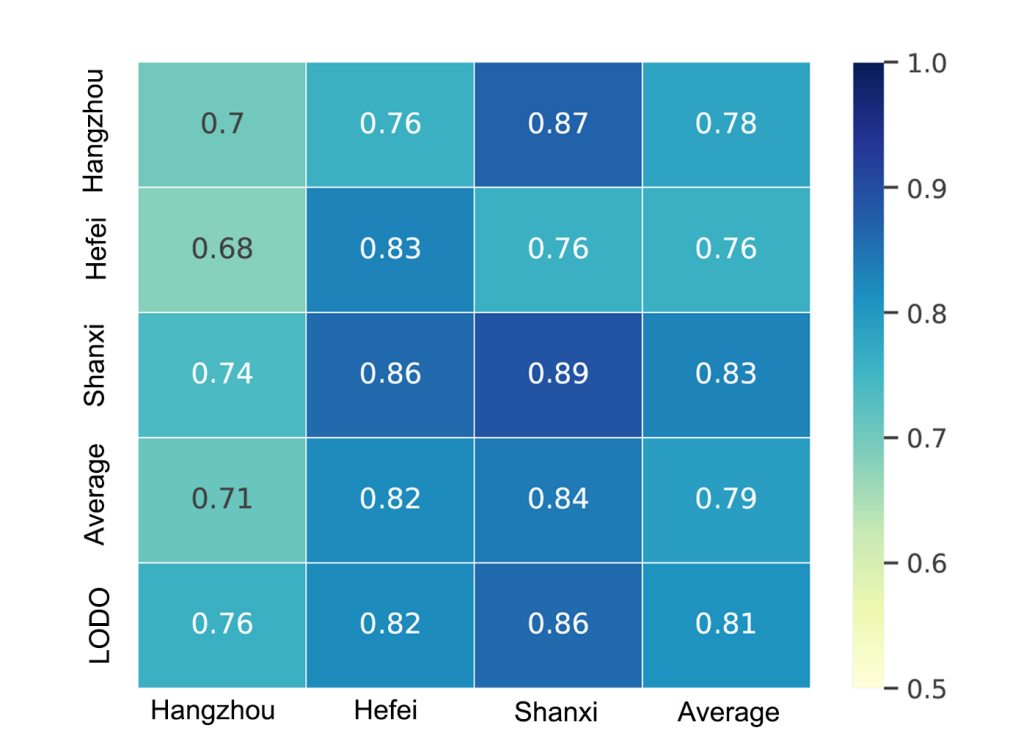


**Supplementary Figure 7.** Study-to-study transfer and LODO validations were used to validate the generalizability of the combination of *Lactobacillus* and *Streptococcus*. In study-to-study transfer validation, the AUC values on the diagonal are the cross-validation results for each study, the off-diagonal values are the AUC values obtained by cross-cohort validation, where the studies in each row are the training set, and the studies in the corresponding columns are the test set. In LODO validation, data from one study is used as a test set to validate the training set consisting of the remaining studies. LODO, leave-one-dataset-out; AUC, area under the curve.

## Supplementary Tables

**Supplementary Table 1.** Literature search strategy

| **Database** | **Search strategy** |
| --- | --- |
| PubMed | ((stomach cancer OR gastric cancer ) AND (microb*) AND (metagenomic or mNGS or 16s rRNA)) NOT Review |
| Web of Science | ALL=(((stomach cancer OR gastric cancer ) AND (microb*) AND (metagenomic or mNGS or 16s rRNA)) NOT Review) |
| EMBASE | ('stomach cancer'/exp OR 'stomach cancer' OR (('stomach'/exp OR stomach) AND ('cancer'/exp OR cancer)) OR 'gastric cancer'/exp OR 'gastric cancer' OR (gastric AND ('cancer'/exp OR cancer))) AND microb* AND ('metagenomic'/exp OR metagenomic OR mngs OR '16s rrna'/exp OR '16s rrna' OR (16s AND ('rrna'/exp OR rrna))) NOT ('review'/exp OR review) AND [19-09-1966]/sd NOT [10-08-2023]/sd |

**Supplementary Table 2.** Methodology for collection of samples included in the meta-analysis.

| **Authors/year** | **Collection method** | **Storage condition** | ***HP* test** | **Excluded population** |
| --- | --- | --- | --- | --- |
| He, 2022 (He et al., 2022) | biopsies were collected during endoscopy | frozen immediately at –80 °C | Yes | age under 40 years; the presence of a serious illness, such as severe cardiopulmonary, renal, or metabolic disease; prior medication history of antibiotics, acid blockers, anti-infammatory agents, or probiotics for past 1 month; prior history of any surgical gastric resection; and refusal of consent to the study |
| Liu D, 2021 (Liu et al., 2021) | biopsies were obtained using forceps from the antrum and the gastric body | frozen immediately after collection within 0.5 h and stored at −80°C | Yes | use antibiotics and probiotics within 1 month and pump inhibitors within 2 weeks |
| Wang Z, 2020 (Wang et al., 2020b) | biopsies of 1–2 mm were obtained using standard gastroscopic forceps | frozen in liquid nitrogen immediately and transferred to the laboratory and stored at −80°C | Yes | taking antibiotics, PPIs, probiotics, prebiotics, chemotherapeutic drugs, and any other drugs affecting gastrointestinal microbiota within the last month; acute or chronic pulmonary, cardiovascular, hepatic, or renal disorders; positive test for human immunodeficiency virus, hepatitis B or C virus; history of major surgery; and pregnant or lactating |
| Wang L, 2020 (Wang et al., 2020a) | antral biopsies were obtained during endoscopic examinations | stored at −80°C until use | Yes | have a history of diabetes mellitus or any other severe complications, including heart, liver, and renal failure; receive antibiotics or proton pump inhibitor treatment 8 weeks prior to the examination |
| Liu X, 2019 (Liu et al., 2019) | tumor and tumor-free tissues were collected and confirmed by pathological diagnosis | flash frozen in liquid nitrogen | Yes | BMI > 30; use of antibiotics, probiotics, prebiotics, or synbiotics in the previous month; preoperative chemotherapy, radiotherapy, or other biological treatment before gastrectomy |
| Coker, 2018 (Coker et al., 2018) | biopsies were obtained from sites of cancer tissues of GC patients | frozen immediately at −80° C | Yes | use antibiotics within 2 months and receive preoperative chemotherapy or radiotherapy prior to the collection of biopsy samples; take proton pump inhibitors for at least 2 weeks before sample collection |
| Yang, 2016 (Yang et al., 2016) | biopsies were obtained from the antrum, incisura angularis, and corpus | frozen in glycerol/thioglycolate and stored at −80°C | Yes | receive proton pump inhibitors, H2-receptor antagonists, or antimicrobials during the 30 day period previous to the endoscopic procedure; major diseases or previous gastrectomy |
| Eun, 2014 (Eun et al., 2014) | biopsies were obtained from the corpus and antrum during upper endoscopy | immediately stored at −80°C | Yes | age under 18 years; the presence of a serious illness such as severe cardiopulmonary, renal, or metabolic disease; prior medication history of antibiotics, acid-blockers, anti-inflammatory agents, or probiotics for past 6 months; prior history of any surgical gastric resection; and refusal of consent to the study |
| Chen C, 2022 (Chen et al., 2022) | fresh fecal samples were collected when patients had not received treatment | frozen at −80℃ | No | complicated blood disease; immune diseases; combination with other tumors; kidney disease, acute/chronic infection, etc.; previous history of gastric cancer; treatment with antibiotics within 1 month |
| Zhang C, 2022 (Zhang et al., 2022) | fresh fecal samples were collected in a special stool tube sterilized internally | Packed in ice packs for transportation to the laboratory and kept at −80°C | No | taking antibiotics or probiotics within 1 month of inclusion; cancer treatment within 3 months of inclusion; presence of other diseases, such as IBS, IBD, and metabolic diseases; lacking the clinical information |
| Qi, 2019 (Qi et al., 2019) | fresh fecal samples were obtained within 2 h after excretion | stored immediately at −80°C | No | other ethnicities except Han Chinese; age under 18 years; exposure to antibiotics, probiotics, immunomodulators and acid blockers for the past month; history of gastrointestinal tract infections; presence of digestive diseases except gastric cancer; presence of type 2 diabetes, autoimmune diseases and other malignant tumors; and history of gastrointestinal tract surgery, chemoradiotherapy, and cholecystectomy |

*HP*, *Helicobacter pylori*; GC, gastric cancer; PPI, proton-pump inhibitor; BMI, body mass index; IBS, irritable bowel syndrome; IBD, inflammation bowel disease.

**Supplementary Table 3.** Method of DNA extraction for samples included in the meta-analysis.

| **Authors/year** | **DNA extraction** | **primer** | **barcode** |
| --- | --- | --- | --- |
| He, 2022 (He et al., 2022) | QIAGEN DNeasy Kit | 515F (5’-GTGCCAGCMGCCGCGGTAA-3’), 806R (5’-GGACTACHVGGGTWTCTAAT-3’) | - |
| Liu D, 2021 (Liu et al., 2021) | low-salt CTAB method | 341F (5’-CCTACGGGNGGCWGCAG-3’), 805R (5’-GACTACHVGGGTATCTAATCC-3’) | - |
| Wang Z, 2020 (Wang et al., 2020b) | QIAamp DNA Mini Kit | F: 5’-GTGCCAGCMGCCGCGGTAA-3’,  R: 5’-GGACTACHVGGGTWTCTAAT-3’ | 6-bp |
| Wang L, 2020 (Wang et al., 2020a) | Qiagen DNeasy blood and tissue kit | 338F/806R | - |
| Liu X, 2019 (Liu et al., 2019) | QIAamp DNA Mini Kit | 319F/806R | - |
| Coker, 2018 (Coker et al., 2018) | QIAamp DNA Mini Kit | 515F (5’-GTGCCAGCMGCCGCGGTAA-3’), 806R (5’-GGACTACHVGGGTWTCTAAT-3’) | - |
| Yang, 2016 (Yang et al., 2016) | modified QIAamp protocol for tissue samples (QIAGEN) | 8F (5’-AGAGTTTGATCCTGGCTCAG-3’),  541R (5’-WTTACCGCGGCTGCTGG-3’) | 8-bp |
| Eun, 2014 (Eun et al., 2014) | Qiagen DNeasy Blood and Tissue Extraction kit | 784F (5’-CGTATCGCCTCCCTCGCGCCATCAG-MID-AGGATTAGATACCCTGGTA-3’), 1061R (5’-CTATGCGCCTTGCCAGCCCGCTCAG-MID-CRRCACCGAGCTGACGAC-3’) | - |
| Chen C, 2022 (Chen et al., 2022) | Guhe Stool Mag DNA Kit | 515F (5’-GTGCCAGCMGCCGCGGTAA-3’), 806R (5’-GGACTACHVGGGTWTCTAAT-3’) | 7-bp |
| Zhang C, 2022 (Zhang et al., 2022) | using the CTAB method | 515F (5’-GTGCCAGCMGCCGCGGTAA-3’), 806R (5’-GGACTACHVGGGTWTCTAAT-3’) | - |
| Qi, 2019 (Qi et al., 2019) | E.Z.N.A.® Stool DNA Kit | 338F (5’-ACTCCTACGGGAGGCAGCAG-3’),  806R (5’-GGACTACHVGGGTWTCTAAT-3’) | - |

CTAB, Cetyltrimethylammonium bromide.

**Supplementary Table 4.** The LEfSe method identified the bacterial taxa that differed most significantly between gastric cancer, benign and normal groups in gastric tissue samples.

| Biomarker_names | Logarithm_value | Groups | LDA_value | P_value |
| --- | --- | --- | --- | --- |
| g__*Actinomyces* | 3.432974267 | Benign | 3.079886552 | 9.60E-46 |
| g__*Corynebacterium* | 3.591108981 | Normal | 3.260390744 | 6.80E-57 |
| g__*Arthrobacter* | 4.23914765 | Gastric_cancer | 3.928635686 | 5.73E-11 |
| g__*Rothia* | 3.344375535 | Benign | 2.951762631 | 2.46E-43 |
| g__*Rhodococcus* | 3.492483626 | Benign | 3.198072997 | 1.34E-68 |
| g__*Propionibacterium* | 4.10132518 | Gastric_cancer | 3.725509913 | 3.66E-22 |
| g__*Tsukamurella* | 3.689332761 | Gastric_cancer | 3.383451324 | 1.16E-10 |
| g__*Bifidobacterium* | 3.945682228 | Normal | 3.669497837 | 5.06E-88 |
| g__*Bacteroides* | 4.368023088 | Normal | 4.0973159 | 1.14E-103 |
| g__*Prevotella* | 4.289748245 | Benign | 3.698685111 | 1.01E-27 |
| g__*Chryseobacterium* | 3.790866781 | Normal | 3.515594781 | 6.55E-56 |
| g__*Anoxybacillus* | 4.069399349 | Gastric_cancer | 3.76646611 | 1.02E-77 |
| g__*Bacillus* | 4.544058182 | Normal | 3.500316184 | 2.78E-25 |
| g__*Geobacillus* | 4.12844578 | Benign | 3.819143527 | 6.48E-18 |
| g__*Virgibacillus* | 3.723295453 | Benign | 3.423038615 | 9.49E-20 |
| g__*Sporosarcina* | 3.481129396 | Benign | 3.183560039 | 5.78E-08 |
| g__*Staphylococcus* | 3.991237907 | Benign | 3.364824524 | 0.007610074 |
| g__*Alloiococcus* | 3.489202366 | Gastric_cancer | 3.197552473 | 1.52E-51 |
| g__*Carnobacterium* | 3.27268679 | Benign | 2.97014634 | 1.17E-21 |
| g__*Granulicatella* | 3.593570573 | Gastric_cancer | 3.302334863 | 4.20E-39 |
| g__*Enterococcus* | 4.37766988 | Normal | 4.035676095 | 1.25E-08 |
| g__*Lactobacillus* | 4.681265276 | Gastric_cancer | 4.331803834 | 8.47E-16 |
| g__*Leuconostoc* | 3.603780037 | Benign | 3.297768099 | 6.88E-15 |
| g__*Lactococcus* | 4.632568459 | Benign | 4.190121948 | 1.62E-10 |
| g__*Streptococcus* | 4.963239407 | Gastric_cancer | 4.37726861 | 2.89E-05 |
| g__*Alkaliphilus* | 3.678914155 | Normal | 3.373928666 | 1.43E-58 |
| g__*Roseburia* | 3.974778204 | Normal | 3.684483763 | 6.72E-64 |
| g__*Peptostreptococcus* | 4.407128844 | Gastric_cancer | 4.071226412 | 5.10E-06 |
| g__*Faecalibacterium* | 4.731390392 | Normal | 4.440079521 | 9.38E-130 |
| g__*Phascolarctobacterium* | 4.1139828 | Normal | 3.825023417 | 4.13E-81 |
| g__*Veillonella* | 3.95241219 | Gastric_cancer | 3.50914571 | 2.72E-07 |
| g__*Parvimonas* | 3.764946352 | Gastric_cancer | 3.416488443 | 2.53E-33 |
| g__*Bulleidia* | 3.448039157 | Benign | 3.070366586 | 9.61E-51 |
| g__*Fusobacterium* | 3.986614411 | Benign | 3.505949781 | 2.00E-19 |
| g__*Bradyrhizobium* | 3.801327607 | Benign | 3.467737191 | 9.71E-86 |
| g__*Ochrobactrum* | 4.84280068 | Gastric_cancer | 4.530347784 | 2.71E-59 |
| g__*Polymorphum* | 3.73811987 | Gastric_cancer | 3.417623771 | 4.57E-18 |
| g__*Salinispora* | 3.453728711 | Gastric_cancer | 3.153524192 | 7.42E-08 |
| g__*Curvibacter* | 3.934157086 | Benign | 3.6353215 | 4.72E-16 |
| g__*Variovorax* | 4.031426827 | Benign | 3.720523309 | 4.73E-07 |
| g__*Cupriavidus* | 4.681847582 | Normal | 4.380807041 | 9.70E-84 |
| g__*Neisseria* | 4.351368599 | Benign | 3.744827093 | 4.68E-58 |
| g__*Citrobacter* | 3.874447409 | Normal | 3.584399675 | 6.77E-66 |
| g__*Enterobacter* | 4.1232211 | Normal | 3.821337018 | 6.13E-94 |
| g__*Escherichia* | 4.233732623 | Normal | 3.827125184 | 1.83E-06 |
| g__*Legionella* | 3.909411347 | Normal | 3.579322199 | 6.27E-60 |
| g__*Actinobacillus* | 3.522128939 | Benign | 2.771982196 | 0.007911488 |
| g__*Haemophilus* | 4.250187851 | Benign | 3.70754284 | 2.06E-63 |
| g__*Acinetobacter* | 4.578318414 | Normal | 4.142581803 | 1.05E-78 |
| g__*Enhydrobacter* | 3.579561378 | Gastric_cancer | 3.235361536 | 4.73E-81 |
| g__*Pseudomonas* | 4.885111858 | Normal | 4.425720058 | 1.10E-14 |
| g__*Thermus* | 3.718525376 | Gastric_cancer | 3.394940793 | 2.57E-30 |

LEfSe, Linear discriminant analysis Effect Size; LDA, linear discriminant analysis.

**Supplementary Table 5.** Predicted functional changes in the KEGG pathway in the gastric microbiota between the gastric cancer and the normal groups.

| Pathway | Logarithm_value | Groups | LDA_value | P_value | Description |
| --- | --- | --- | --- | --- | --- |
| ko00030 | 4.174716308 | Gastric_cancer | 2.998755507 | 6.04E-10 | Pentose phosphate pathway |
| ko00040 | 3.698747234 | Gastric_cancer | 2.615600792 | 0.00000243 | Pentose and glucuronate interconversions |
| ko00051 | 3.886203364 | Gastric_cancer | 2.898102911 | 1.16E-13 | Fructose and mannose metabolism |
| ko00052 | 3.807776155 | Gastric_cancer | 3.048499128 | 2.9E-18 | Galactose metabolism |
| ko00121 | 3.682466159 | Gastric_cancer | 3.206912013 | 0.00000011 | Secondary bile acid biosynthesis |
| ko00130 | 3.920593229 | Normal | 2.921914913 | 1.1E-16 | Ubiquinone and other terpenoid-quinone biosynthesis |
| ko00190 | 3.818937673 | Normal | 2.373889057 | 0.000108918 | Oxidative phosphorylation |
| ko00195 | 3.794585766 | Normal | 2.678954884 | 0.003163821 | Photosynthesis |
| ko00196 | 3.074238891 | Normal | 2.585415144 | 0.00000558 | Photosynthesis - antenna proteins |
| ko00230 | 3.939060999 | Gastric_cancer | 2.611336103 | 0.00000155 | Purine metabolism |
| ko00240 | 4.04567712 | Gastric_cancer | 2.85623323 | 0.00000243 | Pyrimidine metabolism |
| ko00253 | 3.717846734 | Normal | 3.287783674 | 8.74E-20 | Tetracycline biosynthesis |
| ko00260 | 4.05640568 | Gastric_cancer | 2.267552023 | 0.000419296 | Glycine, serine and threonine metabolism |
| ko00281 | 3.960917836 | Normal | 3.2484965 | 0.000012 | Geraniol degradation |
| ko00290 | 4.293160024 | Gastric_cancer | 2.814714281 | 0.00000588 | Valine, leucine and isoleucine biosynthesis |
| ko00300 | 4.162315644 | Gastric_cancer | 2.832876274 | 0.000019 | Lysine biosynthesis |
| ko00350 | 3.679755768 | Normal | 2.703053376 | 0.00048583 | Tyrosine metabolism |
| ko00360 | 3.73731141 | Normal | 2.632363242 | 0.0000264 | Phenylalanine metabolism |
| ko00361 | 3.447806454 | Normal | 2.569660664 | 0.001940126 | Chlorocyclohexane and chlorobenzene degradation |
| ko00362 | 3.667983705 | Normal | 2.617964065 | 0.006365575 | Benzoate degradation |
| ko00363 | 3.402055824 | Normal | 2.5755344 | 0.0000247 | Bisphenol degradation |
| ko00364 | 3.620164046 | Normal | 2.986256884 | 1.55E-10 | Fluorobenzoate degradation |
| ko00380 | 3.761508491 | Normal | 2.639059951 | 0.00070434 | Tryptophan metabolism |
| ko00410 | 3.879504929 | Normal | 2.745794461 | 0.000543249 | beta-Alanine metabolism |
| ko00440 | 3.351661181 | Normal | 2.270577561 | 0.0000079 | Phosphonate and phosphinate metabolism |
| ko00460 | 3.850091209 | Normal | 2.687224958 | 0.00000299 | Cyanoamino acid metabolism |
| ko00471 | 4.331895263 | Gastric_cancer | 3.14607836 | 0.000735133 | D-Glutamine and D-glutamate metabolism |
| ko00480 | 4.057744784 | Normal | 2.828604692 | 3.83E-08 | Glutathione metabolism |
| ko00500 | 3.774012399 | Gastric_cancer | 2.740136935 | 0.000144349 | Starch and sucrose metabolism |
| ko00511 | 3.47729332 | Gastric_cancer | 2.782928386 | 0.000125127 | Other glycan degradation |
| ko00520 | 3.940040065 | Gastric_cancer | 2.875992521 | 0.000000106 | Amino sugar and nucleotide sugar metabolism |
| ko00521 | 4.071419572 | Gastric_cancer | 2.855351117 | 0.00000394 | Streptomycin biosynthesis |
| ko00540 | 4.199841514 | Normal | 3.175359261 | 0.0000122 | Lipopolysaccharide biosynthesis |
| ko00561 | 3.690589821 | Gastric_cancer | 2.416663636 | 2.28E-10 | Glycerolipid metabolism |
| ko00620 | 4.109403845 | Gastric_cancer | 2.621870725 | 0.000000132 | Pyruvate metabolism |
| ko00621 | 3.535874113 | Gastric_cancer | 2.427291977 | 0.00000301 | Dioxin degradation |
| ko00622 | 3.034144558 | Gastric_cancer | 2.105547765 | 0.002296987 | Xylene degradation |
| ko00623 | 3.73478736 | Normal | 2.527646899 | 2.56E-11 | Toluene degradation |
| ko00627 | 3.49207044 | Normal | 2.605115736 | 0.000476423 | Aminobenzoate degradation |
| ko00633 | 3.47472068 | Normal | 2.358202178 | 0.005558715 | Nitrotoluene degradation |
| ko00643 | 3.752516649 | Normal | 3.073510973 | 0.0000169 | Styrene degradation |
| ko00670 | 4.176367913 | Gastric_cancer | 2.763174152 | 0.001227273 | One carbon pool by folate |
| ko00730 | 4.147641583 | Gastric_cancer | 2.934794174 | 0.0000258 | Thiamine metabolism |
| ko00740 | 3.947291402 | Normal | 2.524919974 | 0.000325341 | Riboflavin metabolism |
| ko00760 | 4.051063574 | Normal | 2.244853405 | 0.004250654 | Nicotinate and nicotinamide metabolism |
| ko00770 | 4.216990927 | Gastric_cancer | 2.769896495 | 0.0000883 | Pantothenate and CoA biosynthesis |
| ko00780 | 4.204417303 | Normal | 2.91820842 | 0.00040106 | Biotin metabolism |
| ko00791 | 3.620842279 | Normal | 2.674958141 | 0.00261213 | Atrazine degradation |
| ko00860 | 3.888732378 | Normal | 2.520944342 | 0.002817962 | Porphyrin and chlorophyll metabolism |
| ko00900 | 4.12998589 | Gastric_cancer | 2.772240391 | 0.000548665 | Terpenoid backbone biosynthesis |
| ko00908 | 3.745505551 | Gastric_cancer | 2.530042681 | 0.003630916 | Zeatin biosynthesis |
| ko00910 | 3.865025795 | Normal | 2.59765044 | 9.17E-14 | Nitrogen metabolism |
| ko00920 | 3.953102915 | Normal | 2.685298165 | 5.99E-09 | Sulfur metabolism |
| ko00960 | 3.659775159 | Normal | 2.724750088 | 0.000097 | Tropane, piperidine and pyridine alkaloid biosynthesis |
| ko00965 | 3.16056501 | Normal | 2.833745352 | 0.000000063 | Betalain biosynthesis |
| ko00970 | 4.224794542 | Gastric_cancer | 2.912860285 | 0.006518437 | Aminoacyl-tRNA biosynthesis |
| ko00980 | 3.529369186 | Gastric_cancer | 3.104259341 | 0.000000195 | Metabolism of xenobiotics by cytochrome P450 |
| ko00983 | 3.951672562 | Gastric_cancer | 2.949822228 | 0.00000228 | Drug metabolism - other enzymes |
| ko01040 | 3.813918909 | Normal | 2.617761415 | 0.004595201 | Biosynthesis of unsaturated fatty acids |
| ko01055 | 4.19372433 | Gastric_cancer | 3.281267834 | 1.9E-09 | Biosynthesis of vancomycin group antibiotics |
| ko02020 | 3.764050531 | Normal | 2.82164427 | 1.51E-11 | Two-component system |
| ko02030 | 4.31851384 | Normal | 3.439683596 | 6.36E-08 | Bacterial chemotaxis |
| ko02040 | 4.134182549 | Normal | 3.004152044 | 0.006340414 | Flagellar assembly |
| ko02060 | 3.655474427 | Gastric_cancer | 3.014229173 | 9.6E-12 | Phosphotransferase system (PTS) |
| ko03010 | 4.183717248 | Gastric_cancer | 2.940227044 | 0.001104328 | Ribosome |
| ko03030 | 4.090122038 | Gastric_cancer | 2.815762666 | 0.000226941 | DNA replication |
| ko03070 | 4.085820661 | Normal | 3.101002672 | 3.09E-14 | Bacterial secretion system |
| ko03410 | 3.992083623 | Gastric_cancer | 2.486021668 | 0.000891885 | Base excision repair |
| ko03420 | 3.88463015 | Gastric_cancer | 2.588226832 | 0.001505131 | Nucleotide excision repair |
| ko03430 | 4.20770123 | Gastric_cancer | 2.906380366 | 0.00024256 | Mismatch repair |
| ko03440 | 4.150550365 | Gastric_cancer | 2.874709926 | 0.0000382 | Homologous recombination |
| ko04122 | 4.175071448 | Normal | 2.751690261 | 0.0000087 | Sulfur relay system |
| ko04146 | 3.537893903 | Normal | 2.174189572 | 0.000627988 | Peroxisome |
| ko05111 | 3.523953127 | Normal | 2.774926415 | 4.06E-23 | Vibrio cholerae pathogenic cycle |
| ko05120 | 3.20908886 | Normal | 2.033123963 | 0.0000268 | Epithelial cell signaling in Helicobacter pylori infection |

KEGG, Kyoto Encyclopedia of Genes and Genomes; LDA, linear discriminant analysis.

**Supplementary Table 6.** Characteristics of studies included in the meta-analysis for the *HP*- normal and gastric cancer group and *HP*+ normal and gastric cancer group datasets.

| **No.** | **Authors/year** | **Bioproject** | **Sample Type** | **Country/Region** | **Study Group** |
| --- | --- | --- | --- | --- | --- |
| 1 | He, 2022 (He et al., 2022) | PRJNA481413 | Gastric tissue | China, Nanchang | *HP*- Normal (14)  *HP*- Gastric_cancer (34)  *HP*+ Normal (14)  *HP*+ Gastric_cancer (23) |
| 3 | Wang Z, 2020 (Wang et al., 2020b) | PRJEB26931 | Gastric tissue | China, Beijing | *HP*- Normal (11)  *HP*- Gastric_cancer (31)  *HP*+ Normal (45)  *HP*+ Gastric_cancer (53) |
| 4 | Wang L, 2020 (Wang et al., 2020a) | PRJNA313391 | Gastric tissue | China, Qingdao | *HP*- Gastric_cancer (37)  *HP*+ Gastric_cancer (23) |
| 5 | Liu X, 2019 (Liu et al., 2019) | PRJNA428883 | Gastric tissue | China, Zhejiang | *HP*- Normal (77)  *HP*- Gastric_cancer (72)  *HP*+ Normal (173)  *HP*+ Gastric_cancer (157) |
| 6 | Coker, 2018 (Coker et al., 2018) | PRJNA375772 | Gastric tissue | China, Xi’an | *HP*- Gastric_cancer (5)  *HP*+ Gastric_cancer (14) |
|  |  |  |  | China, Inner Mongolia | *HP*- Gastric_cancer (4)  *HP*+ Gastric_cancer (15) |
| 8 | Eun, 2014 (Eun et al., 2014) | PRJNA239281 | Gastric tissue | South Korea, Hanyang | *HP*- Gastric_cancer (3)  *HP*+ Gastric_cancer (8) |

*HP*-, *Helicobacter pylori*-negative; *HP*+, *Helicobacter pylori*-positive.

**Supplementary Table 7.** The LEfSe method identified the bacterial taxa that differed most significantly between *HP*- gastric cancer and *HP*- normal groups.

| Biomarker_names | Logarithm_value | Groups | LDA_value | P_value |
| --- | --- | --- | --- | --- |
| *g__Arthrobacter* | 3.692897922 | *HP*- Gastric_cancer | 3.402188785 | 7.58E-09 |
| *g__Bifidobacterium* | 3.647345204 | *HP*- Normal | 3.203604071 | 2.61E-13 |
| *g__Bacteroides* | 3.875370842 | *HP*- Normal | 3.529873181 | 1.03E-19 |
| *g__Geobacillus* | 3.352472813 | *HP*- Gastric_cancer | 3.021927412 | 4.77E-06 |
| *g__Enterococcus* | 3.965474397 | *HP*- Normal | 3.559541215 | 4.06E-12 |
| *g__Lactobacillus* | 4.284111802 | *HP*- Gastric_cancer | 3.877019329 | 8.25E-11 |
| *g__Lactococcus* | 3.909623085 | *HP*- Gastric_cancer | 3.42377397 | 0.000652409 |
| *g__Streptococcus* | 4.201603939 | *HP*- Gastric_cancer | 3.73188079 | 0.018692431 |
| *g__Roseburia* | 3.426795829 | *HP*- Normal | 3.107107061 | 3.71E-16 |
| *g__Peptostreptococcus* | 3.672709664 | *HP*- Gastric_cancer | 3.112173625 | 0.005599654 |
| *g__Faecalibacterium* | 4.186929951 | *HP*- Normal | 3.841256654 | 5.25E-12 |
| *g__Phascolarctobacterium* | 3.499405418 | *HP*- Normal | 3.181702889 | 1.69E-14 |
| *g__Fusobacterium* | 3.52740513 | *HP*- Gastric_cancer | 3.044156099 | 0.000503216 |
| *g__Ochrobactrum* | 4.292671478 | *HP*- Gastric_cancer | 3.996873513 | 4.80E-14 |
| *g__Cupriavidus* | 3.539499822 | *HP*- Normal | 3.267154945 | 0.002239636 |
| *g__Neisseria* | 3.530465922 | *HP*- Gastric_cancer | 3.207759118 | 0.003224311 |
| *g__Citrobacter* | 3.54570945 | *HP*- Normal | 3.204874556 | 5.74E-18 |
| *g__Enterobacter* | 3.531225383 | *HP*- Normal | 3.024807553 | 0.0141521 |
| *g__Escherichia* | 3.583266964 | *HP*- Normal | 3.128039418 | 6.27E-13 |
| *g__Acinetobacter* | 3.904768748 | *HP*- Normal | 3.464874886 | 3.26E-09 |
| *g__Pseudomonas* | 4.517914419 | *HP*- Normal | 4.13521477 | 1.30E-15 |

LEfSe, Linear discriminant analysis Effect Size; *HP*-, *Helicobacter pylori*-negative; LDA, linear discriminant analysis.

**Supplementary Table 8.** The LEfSe method identified the bacterial taxa that differed most significantly between *HP*+ gastric cancer and *HP*+ normal groups.

| Biomarker_names | Logarithm_value | Groups | LDA_value | P_value |
| --- | --- | --- | --- | --- |
| *g__Bacteroides* | 3.483209815 | *HP+* Normal | 3.077678549 | 3.80E-08 |
| *g__Enterococcus* | 3.500461457 | *HP+* Normal | 3.022632703 | 2.19E-06 |
| *g__Lactobacillus* | 3.784846988 | *HP+* Gastric_cancer | 3.388513727 | 0.03614964 |
| *g__Streptococcus* | 4.205816455 | *HP+* Gastric_cancer | 3.684051494 | 4.01E-06 |
| *g__Peptostreptococcus* | 3.59059906 | *HP+* Gastric_cancer | 3.089316638 | 0.000532304 |
| *g__Faecalibacterium* | 3.709594026 | *HP+* Normal | 3.217734873 | 2.55E-05 |
| *g__Ochrobactrum* | 3.929122709 | *HP+* Gastric_cancer | 3.604311689 | 0.021254669 |
| *g__Sphingomonas* | 3.750472402 | *HP+* Normal | 3.365105597 | 0.000283688 |
| *g__Cupriavidus* | 4.008006316 | *HP+* Normal | 3.669067761 | 8.69E-06 |
| *g__Pseudomonas* | 3.759670079 | *HP+* Normal | 3.155382695 | 2.07E-09 |

LEfSe, Linear discriminant analysis Effect Size; *HP*+, *Helicobacter pylori*-positive; LDA, linear discriminant analysis.

**Supplementary Table 9.** The LEfSe method identified the bacterial taxa that differed most significantly between gastric cancer and normal groups in fecal samples.

| Biomarker_names | Logarithm_value | Groups | LDA_value | P_value |
| --- | --- | --- | --- | --- |
| *g__Lactobacillus* | 3.313565749 | Gastric_cancer | 2.954701682 | 9.77E-18 |
| *g__Streptococcus* | 3.505458916 | Gastric_cancer | 3.131672246 | 2.47E-05 |
| *g__Clostridium* | 2.850906033 | Normal | 2.402287711 | 0.001760769 |
| *g__SMB53* | 3.518350983 | Normal | 2.938215568 | 0.00494697 |
| *g__Lachnoclostridium* | 2.885256828 | Normal | 2.499718942 | 1.61E-18 |
| *g__Lachnospira* | 3.573206461 | Normal | 3.10757391 | 3.02E-14 |
| *g__Roseburia* | 4.193772121 | Normal | 3.541093983 | 1.49E-08 |
| *g__Faecalibacterium* | 4.473116113 | Normal | 3.736754313 | 2.68E-06 |
| *g__Dialister* | 3.660206435 | Normal | 2.870806267 | 0.001053324 |
| *g__Megamonas* | 3.714524363 | Normal | 3.023443083 | 0.007081427 |
| *g__Phascolarctobacterium* | 3.586507898 | Normal | 2.753086992 | 0.00062438 |
| *g__Sutterella* | 3.082083075 | Normal | 2.37971417 | 7.24E-07 |
| *g__Succinivibrio* | 2.740992451 | Gastric_cancer | 2.410722537 | 0.018351668 |
| *g__Enterobacter* | 2.850455753 | Gastric_cancer | 2.434603151 | 0.000311233 |
| *g__Escherichia* | 4.033866626 | Gastric_cancer | 3.554368996 | 8.36E-06 |
| *g__Klebsiella* | 3.553320235 | Gastric_cancer | 3.067555202 | 0.000366367 |

LEfSe, Linear discriminant analysis Effect Size; LDA, linear discriminant analysis.

**References**

Chen, C., Du, Y., Liu, Y., Shi, Y., Niu, Y., Jin, G., et al. (2022). Characteristics of gastric cancer gut microbiome according to tumor stage and age segmentation. *Appl Microbiol Biotechnol* 106, 6671–6687. doi: 10.1007/s00253-022-12156-x

Coker, O. O., Dai, Z., Nie, Y., Zhao, G., Cao, L., Nakatsu, G., et al. (2018). Mucosal microbiome dysbiosis in gastric carcinogenesis. *Gut* 67, 1024–1032. doi: 10.1136/gutjnl-2017-314281

Eun, C. S., Kim, B. K., Han, D. S., Kim, S. Y., Kim, K. M., Choi, B. Y., et al. (2014). Differences in gastric mucosal microbiota profiling in patients with chronic gastritis, intestinal metaplasia, and gastric cancer using pyrosequencing methods. *Helicobacter* 19, 407–416. doi: 10.1111/hel.12145

He, C., Peng, C., Shu, X., Wang, H., Zhu, Z., Ouyang, Y., et al. (2022). Convergent dysbiosis of gastric mucosa and fluid microbiome during stomach carcinogenesis. *Gastric Cancer* 25, 837–849. doi: 10.1007/s10120-022-01302-z

Liu, D., Chen, S., Gou, Y., Yu, W., Zhou, H., Zhang, R., et al. (2021). Gastrointestinal Microbiota Changes in Patients With Gastric Precancerous Lesions. *Front Cell Infect Microbiol* 11, 749207. doi: 10.3389/fcimb.2021.749207

Liu, X., Shao, L., Liu, X., Ji, F., Mei, Y., Cheng, Y., et al. (2019). Alterations of gastric mucosal microbiota across different stomach microhabitats in a cohort of 276 patients with gastric cancer. *EBioMedicine* 40, 336–348. doi: 10.1016/j.ebiom.2018.12.034

Qi, Y.-F., Sun, J.-N., Ren, L.-F., Cao, X.-L., Dong, J.-H., Tao, K., et al. (2019). Intestinal Microbiota Is Altered in Patients with Gastric Cancer from Shanxi Province, China. *Dig Dis Sci* 64, 1193–1203. doi: 10.1007/s10620-018-5411-y

Wang, L., Xin, Y., Zhou, J., Tian, Z., Liu, C., Yu, X., et al. (2020a). Gastric Mucosa-Associated Microbial Signatures of Early Gastric Cancer. *Front Microbiol* 11, 1548. doi: 10.3389/fmicb.2020.01548

Wang, Z., Gao, X., Zeng, R., Wu, Q., Sun, H., Wu, W., et al. (2020b). Changes of the Gastric Mucosal Microbiome Associated With Histological Stages of Gastric Carcinogenesis. *Front Microbiol* 11, 997. doi: 10.3389/fmicb.2020.00997

Yang, I., Woltemate, S., Piazuelo, M. B., Bravo, L. E., Yepez, M. C., Romero-Gallo, J., et al. (2016). Different gastric microbiota compositions in two human populations with high and low gastric cancer risk in Colombia. *Sci Rep* 6, 18594. doi: 10.1038/srep18594

Zhang, C., Hu, A., Li, J., Zhang, F., Zhong, P., Li, Y., et al. (2022). Combined Non-Invasive Prediction and New Biomarkers of Oral and Fecal Microbiota in Patients With Gastric and Colorectal Cancer. *Front Cell Infect Microbiol* 12, 830684. doi: 10.3389/fcimb.2022.830684
